# Powerful regulatory systems and post-transcriptional gene silencing resist increases in cellulose content in cell walls of barley

**DOI:** 10.1186/s12870-015-0448-y

**Published:** 2015-02-21

**Authors:** Hwei-Ting Tan, Neil J Shirley, Rohan R Singh, Marilyn Henderson, Kanwarpal S Dhugga, Gwenda M Mayo, Geoffrey B Fincher, Rachel A Burton

**Affiliations:** ARC Centre of Excellence in Plant Cell Walls, School of Agriculture, Food and Wine, University of Adelaide, Waite Campus, Glen Osmond, South Australia 5064 Australia; DuPont Agricultural Biotechnology, DuPont Pioneer, Johnston, IA 50131-1004 USA; Adelaide Microscopy Waite Facility, School of Agriculture, Food and Wine, University of Adelaide, Waite Campus, Glen Osmond, South Australia 5064 Australia

**Keywords:** Barley, CaMV 35S constitutive promoter, Cellulose, Gene silencing, *HvCesA* genes, Primary cell walls, Secondary cell walls

## Abstract

**Background:**

The ability to increase cellulose content and improve the stem strength of cereals could have beneficial applications in stem lodging and producing crops with higher cellulose content for biofuel feedstocks. Here, such potential is explored in the commercially important crop barley through the manipulation of cellulose synthase genes (*CesA*).

**Results:**

Barley plants transformed with primary cell wall (PCW) and secondary cell wall (SCW) barley cellulose synthase (*HvCesA*) cDNAs driven by the CaMV 35S promoter, were analysed for growth and morphology, transcript levels, cellulose content, stem strength, tissue morphology and crystalline cellulose distribution. Transcript levels of the PCW *HvCesA* transgenes were much lower than expected and silencing of both the endogenous *CesA* genes and introduced transgenes was often observed. These plants showed no aberrant phenotypes. Although attempts to over-express the SCW *HvCesA* genes also resulted in silencing of the transgenes and endogenous SCW *HvCesA* genes, aberrant phenotypes were sometimes observed. These included brittle nodes and, with the *35S:HvCesA4* construct, a more severe dwarfing phenotype, where xylem cells were irregular in shape and partially collapsed. Reductions in cellulose content were also observed in the dwarf plants and transmission electron microscopy showed a significant decrease in cell wall thickness. However, there were no increases in overall crystalline cellulose content or stem strength in the *CesA* over-expression transgenic plants, despite the use of a powerful constitutive promoter.

**Conclusions:**

The results indicate that the cellulose biosynthetic pathway is tightly regulated, that individual CesA proteins may play different roles in the synthase complex, and that the sensitivity to *CesA* gene manipulation observed here suggests that *in planta* engineering of cellulose levels is likely to require more sophisticated strategies.

**Electronic supplementary material:**

The online version of this article (doi:10.1186/s12870-015-0448-y) contains supplementary material, which is available to authorized users.

## Background

In barley, it is estimated that plant lodging can cause a reduction of up to 65% in grain yield [1]. Weakness in the stem and poor root anchorage, when subjected to external factors such as wind, rain or disease, result in stem/root lodging or the permanent failure of the plant shoot to support its upright position [2]. Stem strength is a complex trait reflecting cellulose content, the length, number and arrangement of vascular bundle fibres in the organ, the orientation of cellulose microfibrils and the degree of lignification [3-5]. These traits contribute synergistically to plant stem strength. Previous studies have shown that a decrease in load-bearing cell wall polymers such as cellulose or lignin can negatively affect stem strength in barley [6], wheat [7], rice [8] and maize [9]. In wheat, a combination of Fourier transform infrared resonance (FTIR) analysis, histology and principle component analysis (PCA), showed that cellulose contributed more to stem strength than lignin [10]. Similarly in maize, Appenzeller *et al.* [11] and Ching *et al.* [9] showed a strong correlation (r^2^ = 0.85) between cellulose content (g/cm) and internodal flexural stem strength, but found no consistent correlation between lignin content and stem strength.

Cellulose content therefore seems to be an important contributing factor in stem strength of cereal species. At the molecular level, cellulose consists of linear, unbranched chains of glucosyl residues linked by (1,4)-β-glucosidic linkages [12]. Cellulose chains are often described as flat ribbons that aggregate into microfibrils of 2 to 2.5 nm in thickness. There is some debate as to the precise number of chains that constitute a microfibril, with values ranging from 36 individual (1,4)-β-glucan chains [13] to as few as 16 chains [14]. The microfibrils can further aggregate to form larger macrofibrils and can serve as a scaffold for the non-covalent cross-linking of other non-cellulosic polysaccharides. In primary cell walls, cellulose microfibrils are generally arranged perpendicular to the axis of cell elongation, although the alignment between microfibrils is not strictly parallel. Such an arrangement of microfibrils provides both strength and flexibility that enable the primary cell walls to withstand turgor pressure and to assist in the cell’s directional growth. In the secondary wall, the microfibrils are more organised and are often aligned in parallel arrays. There can be several layers in secondary walls and within each layer the parallel microfibrils can be oriented at different angles to create laminated layers that further strengthen the wall and restrict the cell’s lateral or radial growth.

Data from transcript analyses in barley are consistent with Arabidopsis mutational studies, insofar as the abundance of *CesA* transcripts in various tissues at different stages of cell wall development, together with co-expression analyses, suggest that two groups of three *CesA* genes are co-ordinately expressed during the growth of the primary cell wall (PCW) and the secondary cell wall (SCW). In barley*, HvCesA1*, *HvCesA2* and *HvCesA6* are believed to be involved in cellulose synthesis during primary cell wall deposition, while *HvCesA4*, *HvCesA7* and *HvCesA8* are postulated to participate in cellulose synthesis during SCW deposition; a total of eight *HvCesA* genes have been identified [15]. It should be noted that these conclusions are based on co-expression of the two groups of three genes and their relatively high transcript levels in tissues that are believed to be undergoing predominantly PCW or SCW deposition. There is no direct evidence in barley that the groups of three enzymes encoded by the three *HvCesA* genes form a multi-enzyme complex, although this seems likely based on data from other systems [16-20].

In the work described here, barley has been transformed with *HvCesA* genes driven by the powerful constitutive CaMV 35S promoter, with a view to increasing cellulose content in the walls of transgenic lines and to evaluating the effects of increased cellulose on stem strength. All three PCW *HvCesA* and two SCW *HvCesA* genes were studied. The *HvCesA5/7* genes were omitted because they appeared to encode enzymes with identical amino acid sequences. The results provide information on the potential for altering cell wall composition in important crop species of the Triticeae from which residual straw, bran from flour milling and spent grain from the brewery might be used in renewable biofuel production.

## Results

### *HvCesA* genes are distributed across the grass genome

**Figure 1:**
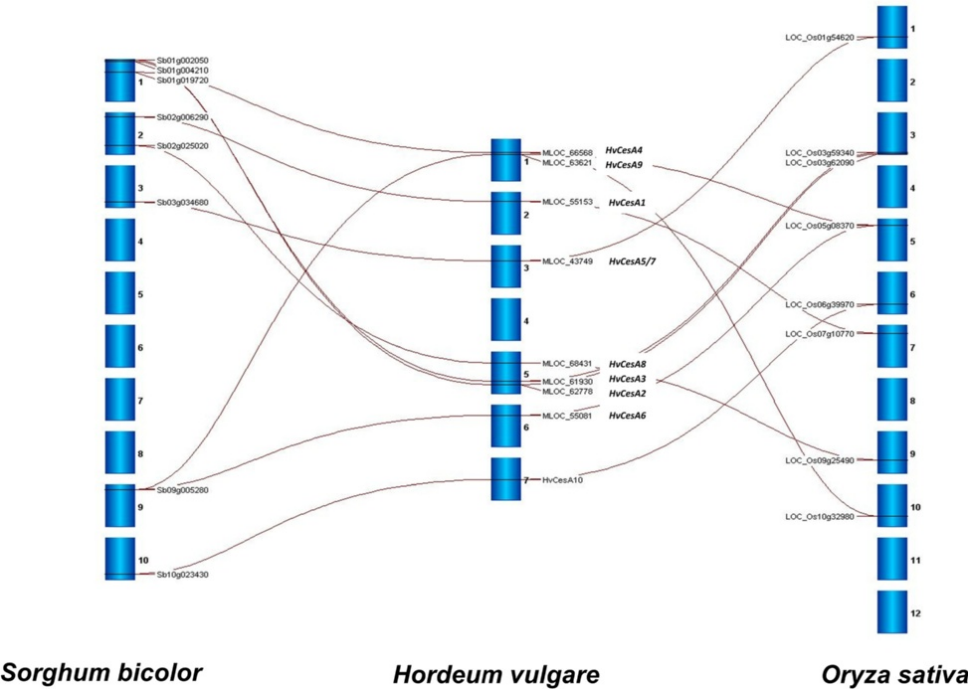
**Image generated using Strudel.** Gray lines show homologous relationships between *CesA* genes in Sorghum (*Sorghum bicolor*), barley (*Hordeum vulgare*) and rice (*Oryza sativa*). Positions of *CesA* genes on the respective chromosomes are also indicated.

At least eight barley (*Hordeum vulgare*) *HvCesA* genes were identified by Burton and co-authors [15]. With the recent release of the barley scaffold [21,22], a total of nine barley *HvCesAs* genes has now been identified. *In silico* mapping of *HvCesA* genes in barley and two other economically important grasses, *Sorghum bicolor* (sorghum) and *Oryza sativa* (rice) indicated that the *CesA* genes are broadly distributed across the genomes, especially so in barley where *HvCesA* genes are found on every chromosome except chromosome 4. Figure 1 shows homologous relationships of the *CesA* genes in barley, sorghum and rice.

### Only plants containing SCW *35S:HvCesA* constructs exhibit aberrant phenotypes

A total of five constructs driven by the CaMV 35S constitutive promoter were individually transformed into barley. These included the three PCW cellulose synthase cDNAs *HvCesA1*, *HvCesA2* and *HvCesA6*, and the two SCW cellulose synthase cDNAs *HvCesA4* and *HvCesA8*. Between 13 and 22 transgenic plants per construct were generated.

**Figure 2:**
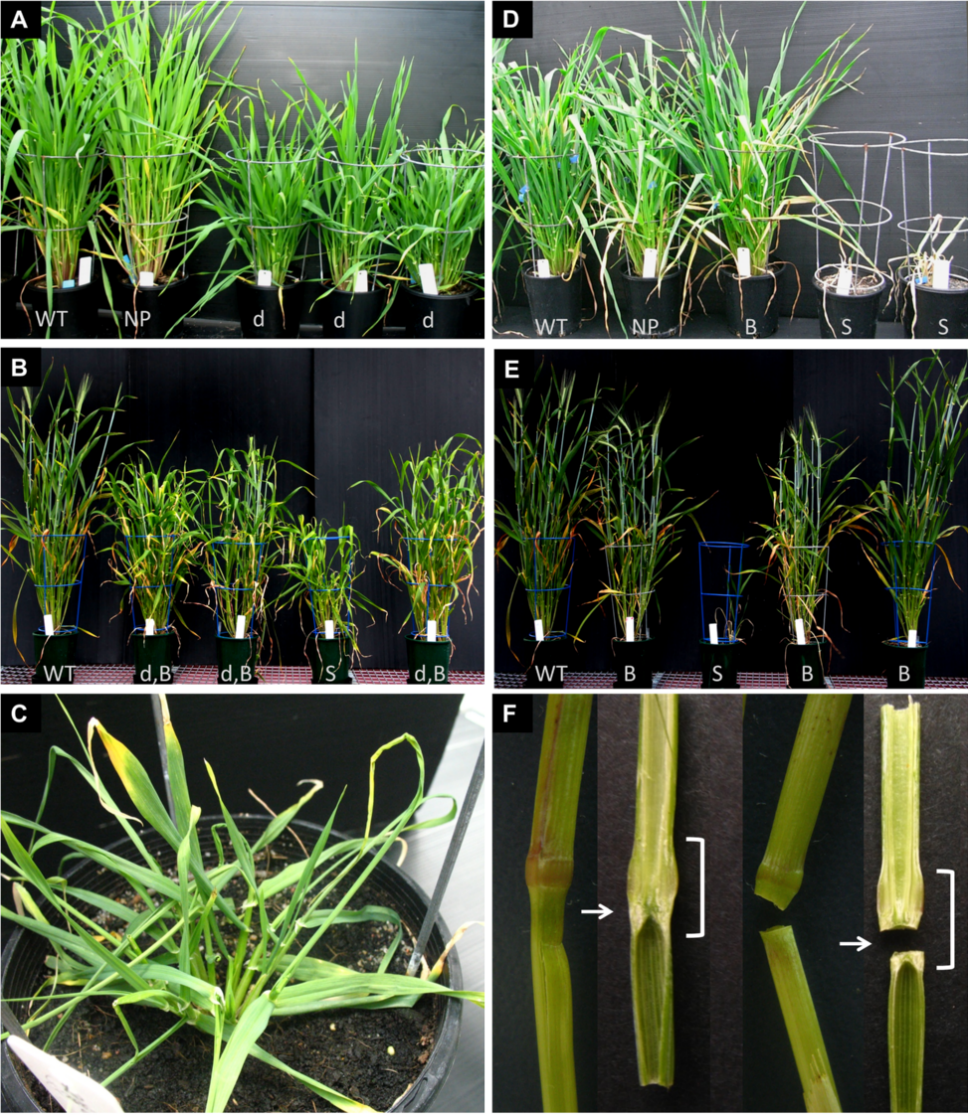
**Photos of representatives from the T**_**1**_ **and T**_**2**_ **generations showing the aberrant phenotypes observed in** ***35S:HvCesA4*** **(A, B,C) and** ***35S:HvCesA8*** **(D,E,F) plants. (A)** T_1_ *35S:HvCesA4* plants and wild-type (WT) Golden promise on the far left. Dwarfism (d) persisted in most plants grown from parents with an aberrant phenotype except for one or two plants within the same line (e.g. plant NP, normal phenotype). The ratio of plants displaying dwarf: normal phenotype (including nulls & revertants) in T_1_ is 58%: 42%. **(B)** Many T_2_, *35S:HvCesA4* progeny were dwarfed with “brittle nodes” (d,B). About 25% of T_2_ plants from each line exhibited a severe reduction in stature, was sterile (S) and some died. The plants with a severe phenotype may be homozygotes. **(C)** Close up view of necrosis found at the leaf-tips of a 1 month old plant that further developed into a dwarf plant with few viable grains. **(D)** T_1_ *35S:HvCesA8* plants. Aberrant phenotypes observed were “brittle node” **(B)** and severely stunted plants that died young (S) (~1 month old). Plants with a “brittle node” phenotype had no reduction in stature but when pressure was applied manually, the stems snapped at the nodes. **(E)** T_2_ *35S:HvCesA8* plants. About 25% of T_2_ plants from each line were stunted and died young (S). Many were only brittle at the node **(B)** with no compromise in stature. **(F)** Comparison of two wild-type (left) and two transgenic “brittle node” stems (right). One stem each from wild-type and transgenic plant were sliced in half to reveal the stem’s internal anatomy. The bracket indicates the nodal region of the stem and at closer inspection the break-point was often found to be at the “nodal plate” (arrow).

Most plants (~90%) transformed with PCW *HvCesA* cDNAs showed no visual abnormalities compared with control Golden Promise barley plants grown under the same conditions. In contrast, more dramatic phenotypes were observed in transgenic plants carrying the SCW *HvCesA* cDNAs. Dwarfism and early-stage leaf necrosis observed in T_0_*35S:HvCesA4* plants persisted into the T_1_ (Figure 2A) and T_2_ generations (Figure 2B). At about one month old, these plants were stunted and necrosis was noticeable at leaf tips (Figure 2C). Dwarf plants took a month longer to reach maturity compared with controls. In the T_2_ generation, about 25% of transgenic progeny from T_1_ dwarf parents either died or were sterile (Figure 2B), suggesting that this severe phenotype might be linked to a homozygous state for the *35S:HvCesA4* gene*.* This could not be directly tested but surviving plants showed evidence of segregation; these plants yielded few viable grains.

For the *35S:HvCesA8* T_1_ and T_2_ generations, most plants showed no obvious difference in height, although the putative homozygotes did not grow past the tillering stage (Zadoks’ scale 22) [23] and subsequently died (Figure 2D and Figure 2E). This is similar to the putative homozygotes found in T_2_*35S:HvCesA4* plants that were stunted and died at an early stage.

Another feature observed in the T_1_ and T_2_ generations of both the *35S:HvCesA4* and *35S:HvCesA8* transgenic plants was brittleness at stem nodes at the heading stage (Figure 2F). This phenotype was apparent for *35S:HvCesA8* in every generation but only became obvious in later generations of *35S:HvCesA4,* especially in the T_2_ generation*.* The break-point of the brittle node phenotype was usually close to the nodal plate but not found within the stem internode as indicated by a horizontal arrow in Figure 2F. There was also a 45% (3.7 mm down to 2.0 mm) reduction in the average stem diameter of dwarf T_1_*35S:HvCesA4* tillers*,* although no significant difference in the diameter of brittle node T_1_*35S:HvCesA8* stems compared with the controls was observed.

### Transcript profiles of T_0_ plants carrying PCW and SCW *35S:HvCesA* constructs

The T_0_ generation of transgenic plants were profiled to determine the effect of PCW and SCW *HvCesA* manipulations. Transcript profiles were generated for sets of transgenic plants carrying the three PCW *HvCesA* cDNAs, namely *35S:HvCesA1*, *35S:HvCesA2* and *35S:HvCesA6* and for the two SCW *35S:HvCesA4* and *35S:HvCesA8* cDNAs*.* For each set of *HvCesA* transgenic plants, transcript levels for the corresponding endogenous genes were also examined (designated *eHvCesA1, eHvCesA2* and *eHvCesA6, eHvCesA4* and *eHvCesA8* [15]). Primers for these endogenous genes are selective and do not amplify the transgene transcript.

Transcript levels for all PCW *HvCesA* transgenes were low with less than 10% of the levels of transcripts for the corresponding *eHvCesA* genes expressed in control plants (Additional file 1: Figures S1A cf. B, S2A cf. C, S3A cf. D and S4A cf.B, C cf. D)*.* Although varying levels of transcript were observed for *eHvCesA1, eHvCesA2* and *eHvCesA6* genes in the three transgenic plant sets, the transcript levels for the endogenous genes were generally lower or equal to those measured in control plants (Additional file 1: Figures S1B-D cf. S2B-D cf. S3B-D and Additional file 1: Table S1). This indicated that both the transgene and endogenous PCW *HvCesA* gene transcript levels were often suppressed in the transgenic lines.

Similarly, the transgene and its endogenous counterpart were profiled in the SCW *HvCesA* T_0_ generation and suppression of endogenous SCW genes was also observed for both constructs (Additional file 1: Figures S4-S5). Unlike PCW transgenic sets, a higher up-regulation of SCW *CesA* transgene levels was observed and measured to be more than 10% of the endogenous levels in control plants. The *HvCesA8* transgene achieved the highest level of up-regulation, measuring 60% of transgene/endogenous ratio (Additional file 1: Table S1).

### Transcript profiles in T_1_ plants containing SCW *35S:HvCesA* constructs

Only subsequent generations of plants carrying SCW constructs were studied, because they exhibited less transgene suppression and hence more likely to have an increased cellulose content. In addition, the observation of drastically distinct phenotypes between the SCW transgenic plants allowed comparisons between the *35S:HvCesA4* and *35S:HvCesA8* constructs.

**Figure 3:**
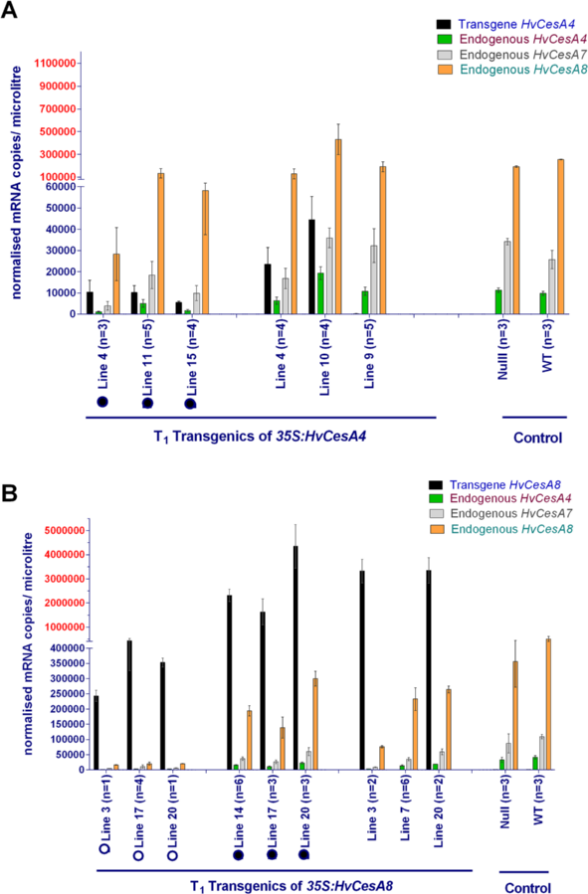
**Averaged transcript levels of four genes in transgenic** ***35S:HvCesA4*** **and** ***35S:HvCes8*** **T**_**1**_ **plants.** X-axis depicts the transgenic lines and control plants (where n = number analysed). The transcript values were averaged for sibling lines with similar phenotype. Where possible, null segregants were selected from three different parental lines. For clarity between very high and low transcript levels, the Y-axis for normalised mRNA copies/microlitre is divided into two different scales (black and red). Error bar is the standard error of the mean (SEM) of biological variation between sibling lines. **(A)** Transcripts measured for SCW *35S:HvCesA4* transgenic plants were the *HvCesA4* transgene and *eHvCesA4*, *eHvCesA7* and *eHvCesA8*. Plants within the same line exhibited variations in phenotype. There were three independent lines with a dwarfed phenotype (black solid circle) and three other with a normal phenotype. **(B)** Transcripts measured for SCW *35S:HvCesA8* transgenic plants were the *HvCesA8* transgene and *eHvCesA4*,*eHvCesA7* and *eHvCesA8*. There were three lines that were stunted, sterile and died young (open circle), three lines with a “brittle node” phenotype (black solid circle) and three lines with a normal phenotype.

Transcript profiles for both SCW transgenic plants showed plants with either aberrant or normal phenotypes, with each phenotype described represented by three independent segregating lines (Figures 3A and 3B). A striking similarity was observed between plants of T_1_*35S:HvCesA4* and T_1_*35S:HvCesA8*, where suppression of endogenous transcript was accompanied by dwarfism. In line with a more severe gene suppression in T_1_*35S:HvCesA8* lines, these plants died early but those in T_1_*35S:HvCesA4* survived to maturity. Dwarfed plants had a lower level of endogenous transcript relative to control and normal-looking plants. Normal plants in T_1_*35S:HvCesA4* showed a two-fold transgene up-regulation and maintained endogenous transcript levels similar to those in control plants (Table 1). However, normal plants in T_1_*35S:HvCesA8* had lower endogenous transcript levels relative to control plants, probably to compensate for the five-fold increase of transgene*.* Detailed transcript profiles for the transgene and the endogenous *eHvCesA4, eHvCesA7* and *eHvCesA8* genes for T_1_ SCW plants relative to control plants are shown in Table 1.

**Table 1:** **Percentage gene levels in transgenic vs control plants**

**Transgenic** ***T***_***1***_ ***35S:HvCesA4***	**Transgene/Endogenous ratio**	**Endogenous** ***HvCesA4***	**Endogenous** ***HvCesA7***	**Endogenous** ***HvCesA8***
dwarf	82%	25%	36%	32%
normal	215%	115%	95%	112%
**Transgenic** ***T***_***1***_ ***35S:HvCesA8***	**Transgene/Endogenous ratio**	**Endogenous** ***HvCesA4***	**Endogenous** ***HvCesA7***	**Endogenous** ***HvCesA8***
stunted	79%	6%	7%	4%
brittle node	644%	44%	42%	14%
normal	518%	32%	35%	44%

Values are calculated as [(T/E)*100] to determine the ratio of transgene transcript levels to its corresponding endogenous gene expressed in control plants, where T = Average gene levels in transgenic plants and E = Average corresponding endogenous gene level in control plants. For endogenous genes, percentage expression in transgenic cf. control plants were calculated.

Another observation in T_1_*35S:HvCesA4* was that, regardless of the phenotypes observed, tight co-regulation between the three endogenous genes was maintained across the whole transgenic set (correlation coefficients, r^2^, of 0.85 to 0.99), indicating that the dwarf phenotype did not perturb the coordination of gene transcription of the three SCW *HvCesA* genes.

In terms of the “tight” co-regulation between the three endogenous *HvCesA* genes, there was a perturbation between *eHvCesA4-eHvCesA8* (r^2^ = 0.2292) and *eHvCesA7-eHvCesA8* (r^2^ = 0.0912) for plants with a ‘brittle node’ phenotype. Co-regulation of *eHvCesA4-eHvCesA7* in the same plants remained tight (r^2^ = 0.8420). For all other plants with either stunted or normal phenotypes, the determination coefficient, r^2^, was in the range 0.53 to 0.86. This was quite different to the dwarfed SCW *CesA* transgenic lines and suggested that the brittle node phenotype may be a direct or indirect result of the perturbed co-regulation between *eHvCesA8* and other *eHvCesA* genes. Furthermore, transcript profiles between normal and brittle node plants in T_1_*35S:HvCesA8* do not differ, which suggests that the aberrant phenotype is not associated with transcript abundance.

### Crystalline cellulose content and stem strength

**Figure 4:**
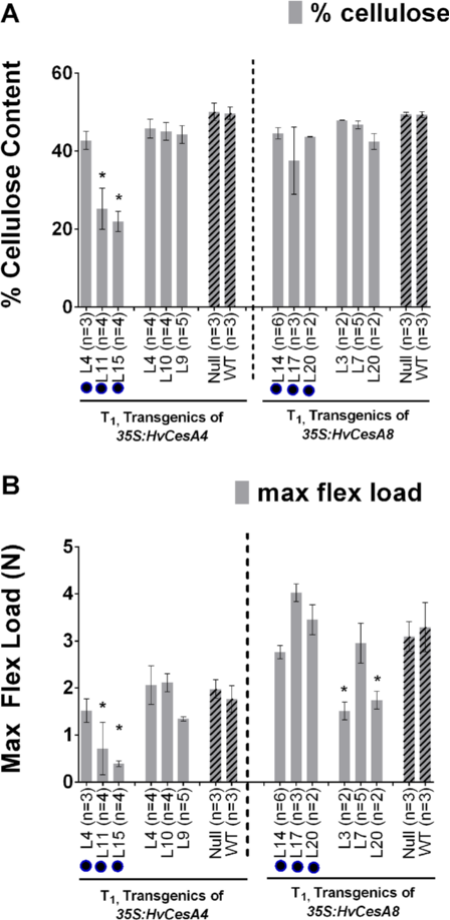
**Cellulose content and stem strength data for T**_**1**_ **SCW 35S:** ***HvCesA4*** **and 35S:** ***HvCesA8*** **plants. (A)** Cellulose content was measured as percent cellulose (%). There were three independent lines with a dwarfed and leaf necrosis phenotype (black solid circle) and three lines with a normal phenotype. **(B)** maximum flexural load, N, was a measure of stem strength. There were three independent lines with ‘brittle node’ phenotype (black solid circle) and three normal-looking transgenic plants. Plants that were severely stunted died at a young stage so were not available for cellulose content analysis. Error bars are standard error of the mean of biological replicates (n). Significant differences were determined by one-way ANOVA followed by *post hoc* Dunnett’s multiple comparisons test.

For T_1_*35S:HvCesA4* plants, there was no significant increase in cellulose content, as measured chemically, whether expressed as % cellulose per total cell wall (Figure 4A) or as mg cellulose per cm stem (data not shown). Normal-looking plants showed a flexural strength similar to the controls plants and most plants with dwarfism displayed a significant reduction in cellulose content and stem strength (Figures 4A and 4B). On average, cellulose per total cell wall decreased by 40% and the stem strength was also reduced to 20% of the average of control plants.

Similarly, no significant increase in cellulose content for the *35S:HvCesA8* T_1_ plants was observed (Figure 4B), even where high levels of transgene transcript were detected, and there was no significant increase in plant stem strength. A 46-53% decrease in stem strength relative to controls was found in Line 3 and Line 20, which are likely due to reason unrelated to cellulose content.

T_0_ plants transformed with constructs PCW or SCW were also analysed and showed that there was no significant increase in either crystalline cellulose content or stem strength (data not shown).

### Observation of crystalline cellulose using immunofluorescent- labelling in stem tissues of T_2_ plants

**Figure 5:**
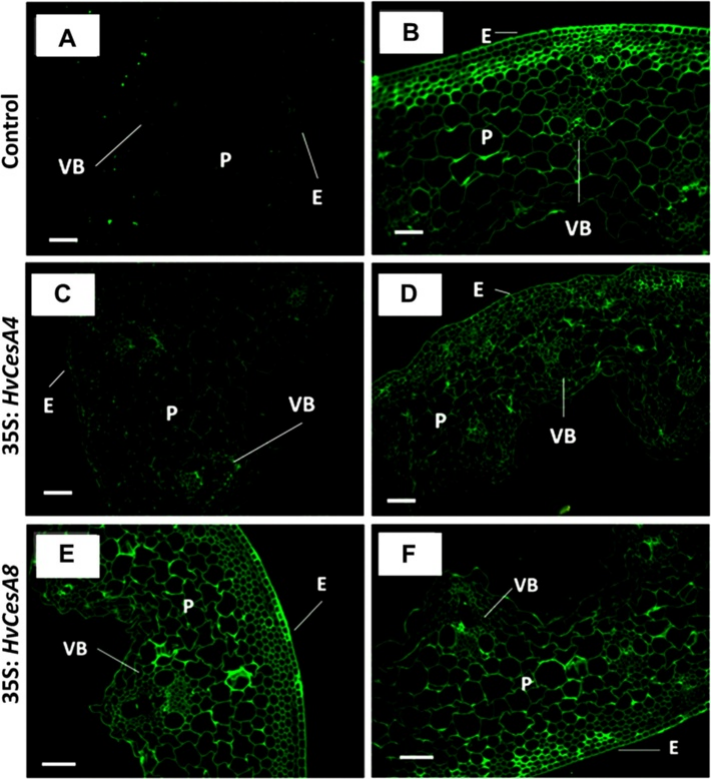
**Immunofluorescent labelling of T**_**2**_ ***35S:HvCesA4*** **and T**_**2**_ ***35S:HvCesA8*** **internode cross-sections. (A)** negative (same treatment as control and transgenic was applied but CBM3a was excluded), **(B)** control = wild type or nulls, **(C)** transgenic *35S:HvCesA4* plant from Line 11, **(D)** transgenic *35S:HvCesA4* plant from Line 15, **(E)** transgenic *35S:HvCesA8* plant from Line 14 and **(F)** transgenic plant from Line 20. Fluorescent images were taken at the same exposure and magnification for all samples. Scale bar is 100 μM. E = epidermis, VB = vascular bundle, PC = parenchyma cell.

To examine potential changes in cellulose distribution as related to the chemically quantitated reduction shown in Figure 4, immunofluorescent labelling with the CBM3a protein was conducted, for both internode and node sections of dwarf T_2_*35S:HvCesA4* plants and brittle node T_2_*35S:HvCesA8* plants (Figure 5).

Fluorescence intensity of labelling on both node and internode sections of T_2_*35S:HvCesA4* was reduced for all cell types (Figures 5C and 5D) compared with control sections (Figure 5B). In the case of the T_2_*35S:HvCesA8* plants (Figures 5E and 5 F)*,* all cell types were labelled at a similar intensity to the control (Figure 5B). Similar reductions in intensity were detected in the node sections for T_2_*35S:HvCesA4* plants (Additional file 1: Figures S6 and S7).

Although the immunocytochemical images will normally give a semi-quantitative estimation of crystalline cellulose, the less intense fluorescence detected in the internode and node sections of T_2_ lines carrying the *35S:HvCesA4* construct was consistent with the reduced amounts of crystalline cellulose measured chemically (Figure 4).

### Tissue architecture, cell wall thickness and lignin distribution in *35S: SCW HvCesA* plants

**Figure 6:**
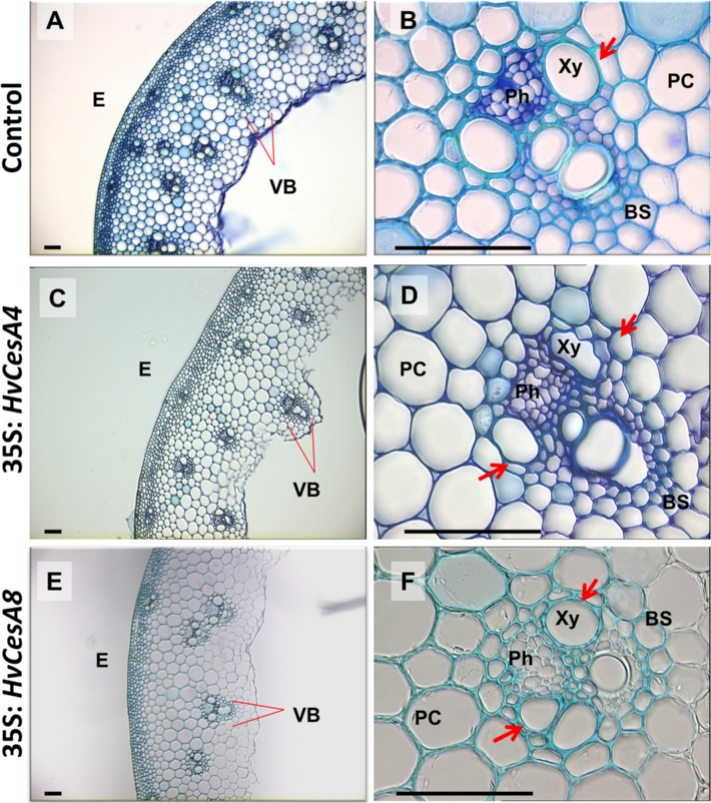
**Light microscopy of cross-sections of T**_**1**_ ***35S:HvCesA4*** **and** ***35S:HvCesA8*** **stem internodes stained with Toluidine Blue.** Equivalent internodes were sectioned using vibratome (~30-50 μM thick) from **(A, B)** wild-type or null, **(C, D)** dwarfed 35S:HvCesA4 transgenic T_1_ plants and **(E, F)** 35S:HvCesA8 transgenic T_1_ plants. Red arrows indicate xylem vessels and in **D**, they are collapsed and irregular in shape. Scale bars denote 100 μM. E = epidermis, VB = vascular bundle, Ph = phloem tissue, Xy = meta-xylem, BS = bundle sheath, PC = parenchyma cells.

Staining with toluidine blue showed that xylem vessels in internode cross-sections of dwarfed *35S:HvCesA4* T_1_ plants were partially collapsed and had irregular boundaries along the elliptical xylem vessels (Figure 6). Collapsed xylem vessels were also observed in leaves from dwarf plants (Additional file 1: Figure S8) but were not seen in *35S:HvCesA8* T_1_ brittle node plants or in control plants, where xylem vessels were round in shape (Figures 6A, 6B, 6E, 6F). For severely stunted *35S:HvCesA8* T_1_ plants, samples were collected and fixed shortly before the plant died. These plants appeared to comprise only the leaves arising from the crown of the base at the plant **(**Additional file 1: Figure S9). Secondary xylem (meta-xylem) did not develop, perhaps because the tissue was too young, but normal proto-xylem development was observed.

**Figure 7:**
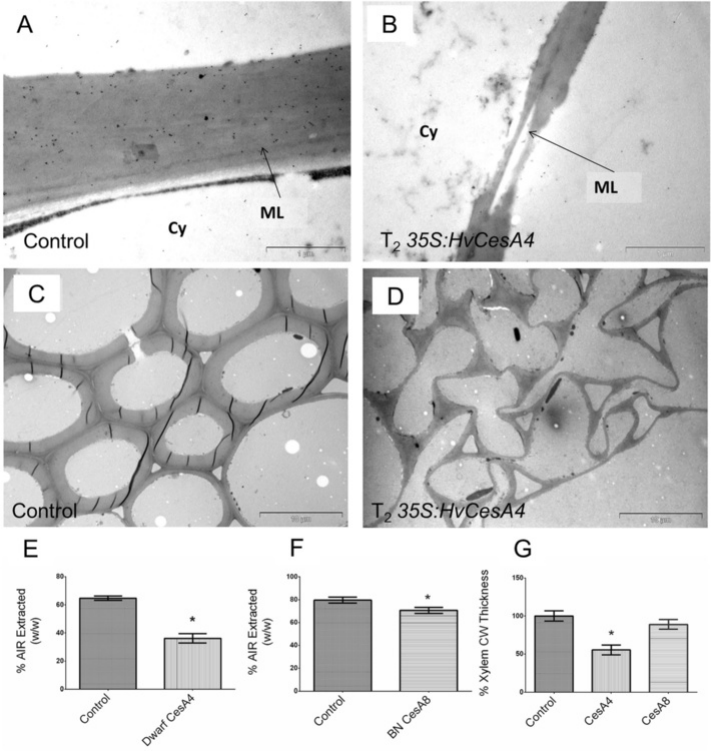
**TEM and measurement of SCW thickening for control and T**_**2**_ **transgenic plants. (A)** Xylem cell wall of WT, **(B)** Xylem cell wall of T_2_ dwarf *35S:HvCesA4,* **(C)** Sclerenchyma cell wall of WT and **(D)** Sclerenchyma cell wall of T_2_ dwarf *35S:HvCesA4*. Scale bar is 1 μM for **(A, B)** and 10 μM for **(C, D)**. Cy = Cytoplasm of bundle sheath cell, ML = middle lamella. **(E, F)** Percent AIR extracted (w/w) from *35S:HvCesA4, 35S:HvCesA8* and control from stem tissues. **(G)** % reduction of xylem cell wall thickness as measured using ImageJ.

Cell walls for collapsed xylem from T_2_*35S:HvCesA4* were examined for reductions in cell wall thickness, as were the sclerenchyma cells (Figures 7A – D). Consistent with the more severe morphology observed in *35S:HvCesA4* dwarf plants (i.e. collapsed xylem)*,* their xylem cell walls were thinner overall, had irregular edges and were occasionally interrupted by apparent gaps in the middle lamella layer. In some cases, two walls detached at the middle lamella (Figure 7B). This was not seen in control plants. The SCW of sclerenchyma cells located under the epidermis of the stem also showed a reduced thickness for both T_2_*35S:HvCesA4* plants (Figure 7D). Cell walls in T_2_*35S:HvCesA4* appeared collapsed and cell wall thickenings were located mainly at cell corners.

Measurements for xylem cell wall thickness were taken from ten images of xylem vessels imaged from two independent lines and a 45% reduction in xylem cell wall thickness was found in plants carrying *35S:HvCesA4* (Figure 7G). This decrease in cell wall thickness was further supported by a decrease in percentage of total cell wall material (AIR) extracted from stem tissues, although we acknowledge that the yield of AIR material will be only semi-quantitative in nature. It was found that dwarf *35S:HvCesA4* and brittle node *35S:HvCesA8* plants had 44% and 10% reductions in total AIR extracted, respectively (Figures 7E, 7F).

Aohara *et al.* [24] attributed a rice “brittle node” phenotype to a drastic reduction of lignified tissues in the node. Nodes from three different T_2_*35S:HvCesA4* and *35S:HvCesA8* plants exhibiting the ‘brittle node’ phenotype were therefore sectioned and stained with phloroglucinol-HCl but no significant changes in lignin content were observed (Additional file 1: Figure S10).

## Discussion

To investigate whether stem strength in barley and hence resistance to lodging might be improved through increasing cellulose levels in cell walls, barley was transformed with individual PCW *(HvCesA1, HvCesA2, HvCesA6)* and SCW (*HvCesA4* and *HvCesA8*) cellulose synthase cDNAs from barley (Burton *et al.*, [15]), driven by the constitutive CaMV 35S promoter. We have used the CaMV 35S promoter successfully to over-express transgenes in barley and other groups have used this promoter to successfully over-express transgenes in rice [25-27], although we acknowledge that alternative promoters such as maize ubiquitin [28] and rice actin [29] have been shown to be generally more active in monocots.

### Visual phenotypes

The *HvCesA* transgenic plants were examined for a visual phenotype, transcript levels of both transgenes and endogenous genes, stem strength, stem morphology, cell wall ultrastructure, cellulose content and crystalline cellulose distribution in the cell wall. More than 90% of T_0_ transgenic plants carrying PCW *35S:HvCesA* constructs exhibited no drastic phenotypic defects but aberrant phenotypes were observed in approximately 25% of the SCW *35:HvCesA* plants and these phenotypes persisted into the T_1_ and T_2_ generations (Figure 2). From the transcript analyses of T_0_ PCW *35S:HvCesA* plants, transgene expression levels for all individuals were very low and in some transgenic populations, the endogenous genes were co-suppressed (Additional file 1: Figures S1-S5). For SCW *35S:HvCesA* plants*,* transgene transcript levels higher than the endogenous transcripts in control plant were found (Figure 3) but this did not result in any significant increase in cellulose content above control levels (Figure 4). Indeed, we were unable to increase the total cellulose content in any of the transgenic lines and in some lines it decreased significantly (Figure 4).

### Reductions in crystalline cellulose

The present work showed that the crystalline cellulose content of *35S:HvCesA4* dwarf plants, as determined by the Updegraff [30] method, was lower than control plants (Figure 4). The reduction in crystalline cellulose was confirmed in both nodes and internodal regions of the stem by immunofluorescence (Figure 5). A reduction in crystalline cellulose may not be the sole contributor to the defect in xylem integrity; a reduction in lignin might also be a contributing factor. Phloroglucinol-HCl staining of xylem cells indicated that although lignin was present, no large differences could be detected between the control and transgenic plants. To quantitate more subtle reductions in lignin content, Klasson lignin assays [31] could be used, but these assays were not applied in the present study.

### Common perturbations in cell morphology

In dwarfed SCW *35S:HvCesA4* barley lines, cell morphology was perturbed and xylem vessels had both collapsed and showed a reduction in cell wall thickness (Figures 6 and 7). A similar phenotype has been described in Arabidopsis *irx* mutants [32]. When Joshi and collaborators [33] introduced another copy of the SCW *Populus tremuloides L* cellulose synthase *PtdCesA8* gene, which is the putative orthologue of barley *HvCesA7*, driven by the CaMV 35S promoter into transgenic poplar plants, severe silencing of both the endogenous and transgene *CesAs*, together with a dramatically reduced cellulose content, dwarfism and a collapsed xylem phenotype, were observed. However, Joshi *et al.* [33] did not report a reduction in wall thickness. In contrast, reductions in cell wall thickness were detected here in T_2_*35S:HvCesA4* plants that exhibited a dwarf phenotype, where the reduction in xylem cell wall thickness was accompanied by an apparent reduction in total extractable cell wall material (Figure 7).

The striking resemblance between phenotypes (collapsed xylem, dwarfism, early leaf senescence) for the *35S:HvCesA4* construct in barley and the cellulose deficient *35S:CesA* transgenic poplar further strengthen the suggestion that, despite the wide phylogenetic distance between a woody tree and a grass, the regulation of SCW *CesAs* may be conserved. However, it is still unclear if the phenotypic changes observed in the barley transgenic lines are directly attributable to silencing the corresponding SCW *HvCesA4* gene or to pleiotropic effects, because mutations other than those in *CesA* genes invoke similar morphological defects. Examples are mutations in genes involved in lignin biosynthesis [34,35], xylan biosynthesis [36], a mutated endoglucanase [37] and pectin biosynthesis via over-expression of QUA2 [38], which all resulted in collapsed xylem vessels.

### Tight regulation and different effects are observed for individual *HvCesA* genes

Our results demonstrated that perturbing *HvCesA* gene expression in the some transgenic lines not only caused extreme phenotypes but also resulted in the silencing of endogenous *HvCesA* genes and, in many cases, in reduced crystalline cellulose contents. It appears likely therefore that barley, and probably other plants, have evolved tight regulatory mechanisms to maintain cellulose levels within a relatively narrow range. Studies in transgenic petunia and other plants indicate that sense co-suppression can be related to promoter strength [39]. However, in the present study, some transgenic lines showed similar or higher endogenous *HvCesA* transcript levels compared with the control plants, but displayed the same phenotypic features as the lines in which transcript levels were lower.

The 35S:*HvCesA4* construct caused more severe developmental defects than the 35S:*HvCesA8* construct. The T_2_ transgenic plants of both constructs were brittle at the nodes but 35S:*HvCesA4* plants were also dwarfed and had collapsed xylem vessels. The differences in the severity of the transgenic phenotypes between 35S:*HvCesA4* and 35S:*HvCesA8* suggest that the protein products of these two secondary cell wall *HvCesA* genes have different or unequal roles in cellulose synthesis. It has been shown that in the *fs2* brittle stem mutant of barley, in which transcription of the *HvCesA4* gene is compromised by the presence of a retrotransposon in the first intron of the gene, cellulose crystallinity is reduced [40]. However, the tight co-regulation between the two groups of three endogenous *HvCesA* genes was not perturbed in the *fs2* brittle stem mutant of barley. In contrast, the tight co-expression of these genes was not always retained in transgenic lines generated in the present study, in which plants with a ‘brittle node’ phenotype showed much reduced co-efficients of determination between *eHvCesA4-eHvCesA8* and *eHvCesA7-eHvCesA8*. Co-regulation of *eHvCesA4-eHvCesA7* in the same plants remained tight (r^2^ = 0.8420). For all other plants with either stunted or normal phenotypes, r^2^ was in the range 0.53 to 0.86. In contrast to the situation in the *fs2* brittle stem mutant [40], the ‘brittle node’ phenotype observed here may be a direct or indirect result of a breakdown of the co-regulation of the *eHvCesA8* gene and genes encoding its putative partners in the cellulose synthase complex.

There is some evidence of redundancy and dual functionality in the roles of CesA proteins in Arabidopsis, where the PCW AtCesA2 and AtCesA5 proteins are partially redundant to AtCesA6, one of the three CesA protein subunits in the PCW cellulose synthase complex [18,41,42]. Moreover, the Arabidopsis SCW AtCesA7, which is the putative orthologue of the barley SCW HvCesA8, was found to partially rescue defects in the PCW mutant *cesa3* and, conversely, PCW *AtCesA1* was able to partly rescue defects in the SCW mutant *cesa8ko* [43]. This suggests that there is flexibility between CesA protein function depending on the tissue or environmental conditions [44]. It might also be argued that the constitutive expression of the 35S promoter leads to a negative dominant phenotype by disturbing the endogenous gene expression in the secondary wall forming cells. Mis-assembly of the functional enzyme complex may ensue because of disturbed stoichiometry of the various subunits. The transcript profiles from this study also showed that stoichiometry is maintained, pointing to the existence of a homeostatic mechanism. The *HvCesA8* gene showed strong overexpression in most lines and was associated with the downregulation of endogenous genes in lines with dwarf phenotypes. The brittle phenotype was correlated with high transgene expression in the absence of strongly reduced transcript levels of endogenous *CesA* genes. This suggests that the perturbation of the stoichiometry of individual CesA proteins in the cellulose synthase complex (CSC) may be responsible for the brittle phenotype.

### Brittle stems arise by different genetic lesions

In the *fs2* barley mutant, a brittle stem phenotype arose due to the insertion of a retroelement in the first intron of the *HvCesA4* gene. This *fs2* mutant had reduced crystalline cellulose and increased non-crystalline cellulose compared to control plants [40]. This showed that *HvCesA4* is essential for the integrity of the cell wall that would otherwise lead to brittleness of the stem. In the present study, brittleness caused by SCW *35S:HvCesA4* was restricted to the node region of the plant stem, unlike the brittleness found in the *fs2* mutant [40]. The less severe phenotype may arise due to the presence of low level of functional CesA4 protein in the silenced transgenic lines described here, whereas in the *fs2* mutant, there may have been no functional CesA4 protein present. As to why the phenotype was restricted to the nodal region, perhaps these joints are the weakest point of the stem and any cell wall defects would be more obvious in this area.

The “brittle node” phenotype exhibited by both *35S:HvCesA4* and *35S:HvCesA8* plants also resembled the phenotype previously described in the rice *bc5* brittle culm mutant [24]. The exact gene affected by this mutation was not identified but SCW *OsCesA* transcripts were suppressed and it was found that walls of sclerenchyma cells in the leaf sheath bundle around the stem node were thinner [24]. Although suppression of all SCW *eHvCesA* genes was also found in the “brittle node” plants in the present study, gene suppression alone cannot explain the “brittle node” phenotype observed in barley because other transgenic plants with a normal phenotype displayed similar suppression of transcripts. The “brittle node” phenotype may be caused by the disrupted coordination between HvCesA4 and HvCesA8 proteins as a result of altered transcript levels, although other factors such as the formation of the rosette complex, cellulose assembly and interactions with other players in cell wall assembly are also likely to affect the final phenotype of a plant.

Our inability to increase cellulose content in the transgenic barley lines may also be attributable to a requirement for all three *HvCesA* genes in a complex to be over-expressed simultaneously. Thus, concurrent up-regulation may be needed to produce a successful increase in cellulose. Although attempts to simultaneously up-regulate the three co-ordinately expressed PCW and SCW *HvCesA* genes were not within the scope of this paper, such a goal using the current lines is possible in the future.

## Conclusions

In summary, the results presented here indicate that potential challenges could be encountered in attempts to engineer cellulose levels *in planta* by manipulating either PCW or SCW *HvCesA* genes using the CaMV 35S promoter. However, the observed pleiotropic phenotypes and transcript silencing arising from our systematic introduction of individual *HvCesA* genes into transgenic barley provided us with an opportunity to deduce the roles of individual *HvCesA* genes. Our results demonstrated unequal roles within SCW genes and between PCW and SCW genes to maintain the structural integrity of cell walls and on the overall ability of the plants to stay upright. The *HvCesA4* gene showed the most negative effects on plant growth in barley. Similar developmental defects observed by Joshi and colleagues [33] for a tree species indicate tight regulation of cellulose biosynthetic genes occurs across the plant kingdom and further work is needed to unravel the complexity of this process.

## Methods

### *In Silico* mapping of *CesA* genes in grasses

Barley, rice and sorghum *CesA* gene positions were estimated from the barley scaffold genome sequence [22,45] and Ensembl Plants (http://plants.ensembl.org/info/website/ftp/index.html) [46] and mapped using Strudel, a stand-alone Java desktop application that allows the simultaneous multi-way comparison of several genomes (http://bioinf.scri.ac.uk/strudel/; [47]).

### Vector construction

Three PCW and two SCW *CesA* genes were individually cloned into the pMDC32 vector [47]. The forward and reverse primer pairs used to clone each full length gene are given in Table S1. Full length *HvCesA1*, *HvCesA2*, *HvCesA4, HvCesA6* and *HvCesA8* cDNAs were generated as described in Burton and co-authors [15] and were cloned into the pCR8®/GW/TOPO TA vector (Life Technologies, Australia). Clones from each construct were digested with restriction enzymes to select those with a sense orientation and were subsequently sequenced on an ABI 3700 (Applied Biosystems Inc., Australia) at the Australian Genome Research Facility (Adelaide, Australia) to verify the identity of genes and the precision of constructs. Each *HvCesA* cDNA was transferred (Life Technologies, Australia) into a Gateway-enabled constitutive expression vector, pMDC32 [48], carrying dual 35S promoters and a NOS terminator that flank the inserted *CesA* cDNA at the 5′ and 3′ ends of the gene, respectively.

### Barley transformation and plant material

A total of five constructs were individually transformed into *Agrobacterium tumefaciens* strain AGL-1, using the freeze-thaw method [49]. The procedures used to grow barley donor plants (*Hordeum vulgare* cv. Golden Promise) and prepare immature scutella for transformation were previously described in Burton *et al.* [25]. The scutella were cultured on callus induction medium in the dark at room temperature for a day prior to transformation. The constructs were transformed into barley using the protocol developed by Tingay *et al.* [50] and modified by Matthews *et al.* [51].

The T_0_ and T_1_ transgenic plants were grown under standard glasshouse conditions as described in Burton and colleagues [15] while T_2_ plants were grown in The Plant Accelerator (Australian Plant Phenomics Facility) under the same conditions. Transgenic plants of T_1_*35S:HvCesA4* and *35S:HvCesA8* were grown at different times of the year, thus direct comparison of transcript and cellulose data are made only between transgenic and control plants. Transgenic plants and their comparable control plants were grown in the same growth chamber and the control plants were part of a random design.

Fertile T_0_ transgenic plants, which exhibited either normal or aberrant phenotypes, and further contained a low locus number of the transgene (one to two), were selected to grow on to the T_1_ and T_2_ generations. The presence of the transgene in T_1_ and T_2_ plants was confirmed using a Phire® Plant Direct PCR Kit (Finnzymes, Vantaa, Finland) and melt curve PCR, respectively. In addition, random T_1_ transgenic plants were selected for Southern hybridisation to verify the presence of the transgene (data not shown).

### RNA isolation, cDNA synthesis and transcript analysis

Total RNA was extracted from leaves of 6-week-old barley (cv Golden Promise) using a commercially prepared guanidine reagent, TRIzol (Life Technologies, Australia) following the manufacturer’s instructions. All RNA samples obtained were treated with the TurboDNA-*free* DNAse kit (Ambion) to remove genomic DNA contamination. The procedure used for cDNA synthesis was that described by Burton *et al.* [15].

Real time quantitative PCR (QPCR) amplification of the 3′ untranslated region (UTR) of *HvCesA* genes was conducted with gene-specific primers as described in Burton *et al.*[15]. Primers used to detect transgenes are listed in Additional file 1: Table S2. It was possible to distinguish between endogenous and transgene *HvCesA* DNA because the 3′UTR of the transgene was shorter and lacked the endogenous reverse primer binding site. The reverse primer for the transgene was designed to hybridize to the NOS terminator, which is not present in a wild-type gene (Additional file 1: Figure S6). The amplified QPCR products were subsequently sequenced to verify the identity of the gene fragment. QPCR was carried out as in Burton *et al.* [52] and the cDNA population used for the initial amplification to determine the acquisition temperatures of QPCR products (Additional file 1: Table S2) was a mixed population of cDNAs synthesized from a cocktail of RNAs extracted from different transgenic plants.

The same control cDNAs were used for QPCR analysis of all five sets of T_0_ transgenic plants. Control cDNAs for T_1_ SCW *35S:HvCesA* plants were null and wild type plants were sown together with the transgenic plants. As a control for non-specific binding, Q-PCR was conducted to detect transgene transcript levels in wild type cDNAs but only background levels were observed.

### Microscopy

Stem sections of at least three T_1_ plants from each line of plants transformed with *35S:HvCesA4* or *35S:HvCesA8* were fixed in freshly prepared Farmer’s fixative (ethanol:acetic acid = 3:1) for 24 hours and transferred to 70% ethanol at 4°C. For all plants, the second stem internode below the flag leaf was sectioned, except for stunted plants that died before proper stem development.

For general morphological studies, fixed internode tissues were briefly rinsed with water and sectioned with a Leica VT1200 vibratome (30–50 μm thickness, 0.4 mm/s velocity, 0.4 mm amplitude). Sections were stained with 0.05% toluidine blue in benzoate buffer pH 4.4, for 2 min and rinsed with water seven times. To obtain uniform sections for soft stems (plants that died young) and ensure comparability, all stem pieces from *35S:HvCesA8* lines were further dehydrated through a series of ethanol concentrations, embedded in paraffin wax and sectioned on a RM 2155 Microtome (Leica) to a thickness of 7 μm. After dewaxing and rehydration, the sections were stained with 0.05% toluidine blue.

To examine lignin, Farmer’s fixed sections of nodes and internodes were stained with 2% (w/v) phloroglucinol (Sigma) in 95% ethanol, followed by a water rinse before treatment with hydrochloric acid. Experiments were carried out as described in Liljegren [53]. All images were captured on Leica AS LMD Laser Dissection Microscope.

### Stem strength measurement

Five stems per plant were tagged during plant development. Stems used for measurements were from the five tallest tillers from the plant. The primary tiller was not used because it is usually the strongest and therefore is different in strength to the secondary tillers. The barley transgenic T_0_ and T_1_ lines were grown in a glasshouse until maturity and air dried stem samples (7-8% moisture content) were used for stem strength measurements. An Instron 5543 materials testing instrument was used to test for three-point flexural strength at a span distance of 40 mm and an anvil rate of 60 mm min^−1^ and data were analysed using Bluehills 2 material testing software. Measurements for flexure load at break (N) were taken at the third internode below the flag leaf. The flexural load (N) required to bend the midpoint of each internode was recorded. The average flexure load for three out of the five internodes with the most similar diameters was calculated. Other measurements such as stem diameter and internode length were also recorded. All transgenic, null and wild type plants were tested.

### Cell wall preparations and crystalline cellulose assay

Freeze dried stems from segments of three internodes directly below the flag leaf were ground to a powder using a Spex 2000 GenoGrinder [2–5 min at 500 (1500strokes/min)] and alcohol-insoluble residues (AIR) were extracted in accordance with Zhang and co-authors [54]. A modified Updegraff (acetic/nitric acid cellulose assay) [30] method, described in Burton and co-authors [40], was performed to determine crystalline cellulose content (% stem or cell wall weight) and mg of cellulose per cm stem. Cellulose assay for all T_1_ transgenics and controls were determined for AIR (alcohol insoluble residues) material. All available T_1_ plants were tested with at least three independent lines for each construct. Cellulose value for each plant was calculated from measurements obtained from three separate tillers per plant. For each cellulose assay, coefficient of variance for technical replicates was less than 15%.

### Immunofluorescent labelling with CBM3a by light microscopy

Barley stem sections (1 cm internode and node) from T_2_ 35S:*HvCesA4* and 35S:*HvCesA8* plants were fixed overnight at 4°C in 4% sucrose, 4% paraformaldehyde and 0.25% glutaraldehyde. Samples were washed with phosphate buffered saline (PBS), pH 7.4 and dehydrated in an increasing series of ethanol concentrations (70%, 80%, 90% 95%, 100%) and finally embedded in LR white resin (ProSciTech, Australia). Sections of 1 μm thickness were cut on a Leica Ultracut R microtome using a diamond knife and affixed onto Poly-L-Lysine glass microscopy slides (Thermo Scientific, Australia). A three-stage immunolabelling method was conducted as described in McCartney [55] with the following modifications optimised for barley stem sections. Specimens were incubated with 6.25 μg/mL CBM3a (Plant Probes, Leeds, UK) [56] in 1% bovine serum albumin/phosphate-buffered saline (BSA/PBS), pH7.4 for 1 h, followed by a 1:100 dilution of the mouse anti-Histidine (His) monoclonal antibody (Sigma-Aldrich, Australia) for 1.5 h and a 1:100 dilution of Alexa Fluor 488 goat anti-mouse IgG (Life Technologies, Australia) for 1 h in the dark. Samples were washed with PBS, mounted in 90% glycerol and viewed on a Leica AS LMD Laser Dissection Microscope with a DFC 480 camera, using fluorescence filter I3 (excitation filter 450–490 nm BP, barrier filter 515 nm LP). For each construct at least three plants from two independently transformed lines were imaged.

In control experiments, the specimen was incubated with a combination of mouse anti-His monoclonal antibody and Alexa Fluor 488 goat anti-mouse IgG, while the CBM3a protein was omitted. Results from the immunofluorescent labelling are comparable because cross-sections from control and transgenic plants were treated on the same slide under the same conditions. At least two plants from two independent lines were examined and all images were captured at the same exposure.

### Transmission Electron Microscope (TEM)

Two plants, each from a single independent line, were examined for each transgenic construct. Null and wild type plants were examined as controls. Sample preparation was as described for immunofluorescent labelling except sections of 80 nm were cut and collected on coated nickel grids (200 mesh parallel). Grids were stained with 2% (w/v) aqueous uranyl acetate (Sigma-Aldrich, Australia) before images were taken using a Philips (Eindhoven, The Netherlands) BioTwin transmission electron microscope and a Gatan multiscan digital camera. The mean of cell wall thickness for each construct was estimated from 10 images representing xylem cells from two different independent lines using ImageJ [57].

### Statistical analysis

All statistical analyses such as ANOVA and correlation analysis were performed using GraphPad Prism (GraphPad Software, Inc., California).

### Availability of supporting data

Sequence data from this article can be found in the GenBank data libraries under accession numbers [AY483150: *HvCesA1*, AY483152: *HvCesA2*: HM222644: *HvCesA4,* AY483155: *HvCesA6* and KM45970: *HvCesA8*]*.* All the supporting data are included as additional files.

## Additional file

Additional file 1: Table S1. Ratios of transgene/endogenous control levels and the percentage reductions of endogenous transcript levels (average value). **Table S2.** Primers used for cloning *HvCesA* full length genes with 5′ and 3′ends. **Table S3.** QPCR primers for transgene. **Figure S1.** Box plot graphs showing transcript profiles for all *35S:HvCesA1* plants. **Figure S2.** Box plot graphs showing transcript profiles for all *35S:HvCesA2* plants. **Figure S3.** Box plot graphs showing transcript profiles for all *35S:HvCesA6* plants. **Figure S4.** Box plot graphs showing transcript profiles for all *35S:HvCesA4* plants. **Figure S5.** Box plot graphs showing transcript profiles for all *35S:HvCesA8* plants. **Figure S6.** Immunofluorescent labelling of T_2_ *35S:HvCesA4* node cross-sections. **Figure S7.** Immunofluorescent labelling of T_2_ *35S:HvCesA8* node cross-sections. **Figure S8.** Partially collapsed xylem vessels found in vibratome sections. **Figure S9.** Light microscopy of cross-sections of *35S:HvCesA8* stem internodes stained with Toluidine Blue. **Figure S10.** Bright field microscopy of phloroglucinol-HCl stained node and internode cross-sections. **Figure S11.** Schematic representation of the primer binding sites for endogenous and transgene HvCesAs.

## Abbreviations

*CesA*: Cellulose synthase genes
PCW: Primary cell wall
SCW: Secondary cell wall
*HvCesA*: Barley cellulose synthase
FTIR: Fourier transform infrared resonance
PCA: Principle component analysis
CBM3a: Crystalline cellulose-binding module (recombinant CBM protein)
QPCR: Real time quantitative PCR
AIR: Alcohol insoluble residues
PBS: Phosphate buffered saline
