# Supplementary material for: Powerful regulatory systems and post-transcriptional gene silencing resist increases in cellulose content in cell walls of barley
Source: BMC Plant Biol. 2015 Feb 21;15:62. doi: 10.1186/s12870-015-0448-y (PMC4349714; doi:10.1186/s12870-015-0448-y)
Supplement: Additional file 1: Table S1. — Ratios of transgene/endogenous control levels and the percentage reductions of endogenous transcript levels (average value). Table S2. Primers used for cloning HvCesA full length genes with 5′ and 3′ends. Table S3. QPCR primers for transgene. Figure S1. Box plot graphs showing transcript profiles for all 35S:HvCesA1 plants. Figure S2. Box plot graphs showing transcript profiles for all 35S:HvCesA2 plants. Figure S3. Box plot graphs showing transcript profiles for all 35S:HvCesA6 plants. Figure S4. Box plot graphs showing transcript profiles for all 35S:HvCesA4 plants. Figure S5. Box plot graphs showing transcript profiles for all 35S:HvCesA8 plants. Figure S6. Immunofluorescent labelling of T2 35S:HvCesA4 node cross-sections. Figure S7. Immunofluorescent labelling of T2 35S:HvCesA8 node cross-sections. Figure S8. Partially collapsed xylem vessels found in vibratome sections. Figure S9. Light microscopy of cross-sections of 35S:HvCesA8 stem internodes stained with Toluidine Blue. Figure S10. Bright field microscopy of phloroglucinol-HCl stained node and internode cross-sections. Figure S11. Schematic representation of the primer binding sites for endogenous and transgene HvCesAs. [file 12870_2015_448_MOESM1_ESM.docx]

**Additional file 1**

**S**upplemental information contains 3 Supplemental Tables and 11 Supplemental Figures.

**Table S1**  Ratios of transgene/endogenous control levels and the percentage reductions of endogenous transcript levels (average value).

| **Transgenic Set (x)** | **Transgene/Endogenous**  **Ratio** | **Endogenous *HvCesA1*** | **Endogenous *HvCesA2*** | **Endogenous *HvCesA6*** |
| --- | --- | --- | --- | --- |
| *T_0_ 35S:HvCesA1* | <10% | N/S | N/S | 48% * |
| *T_0_ 35S:HvCesA2* | <10% | N/S | 80% * | 92% * |
| *T_0_ 35S:HvCesA6* | <10% | N/S | 37% * | 40% * |
| **Transgenic Set (x)** | **Transgene/Endogenous**  **Ratio** | **Endogenous *HvCesA4*** | **Endogenous *HvCesA8*** |  |
| *T_0_ 35S:HvCesA4* | 17% | 95%* | - |  |
| *T_0_ 35S:HvCesA8* | 60% | - | 72% * |  |

Values are calculated as [(T/E)*100] to determine the ratio of transgene transcript levels to its corresponding endogenous gene expressed in control plants, where T = Average transgene level and E = Average corresponding endogenous gene level. To calculate percentage reductions, the formula used was: [(wt-tx)/wt*100]; wt = Average wild-type transcript values; tx = Average transgenic transcript values. Values shown to be significant (p<0.05) by Student’s t-test are indicated by (*). N/S denotes no significant diference from the wild-type averaged transcript values

**Table S2** Primers used for cloning *HvCesA* full length genes with 5’ and 3’ends.

| **Gene** | **Forward Primer 5’>3’** | **Reverse Primer 5’>3’** |
| --- | --- | --- |
| ***HvCesA1*** | AGGAGCCGCTCCAGCTCGGTT | **GTGGATCCGAAGCCCTGACC**CGTAGTAATTCGCAACCCCAAAC |
| ***HvCesA2*** | CCTCCTTCGAGAGAGTCTGAGC | **GCATTAACCCTCACTAAAGCCC**ACTGACTTGCTGGCTGGCTGT |
| ***HvCesA6*** | CTCGACTGAAGCGAGCGAGAGG | **GATGGTCCTGCTGGAGTTCAC**CATGGGGCGACTAACAAAGGGGCA |
| ***HvCesA4*** | CCCTCCTCCACCACATCATCA | **GTGGATCCGAAGCCCTGACC**GATTATACAATGCCCCAAAAGTGC |
| ***HvCesA8*** | CATGGAAGCGGGCGCCGG | **GATGGTCCTGCTGGAGTTCAC**CTTGTTTAAACCATTTCTGGCC |

Additional tags were also fused to reverse primers for each gene (in bold) to enable identification of each construct if transgenic plants were to be crossed.

**Table S3** QPCR primers for transgene

| **Transgene** | **Forward Primer 5’>3’** | **Reverse Primer 5’>3’** | **PCR size (bp)** | **Acquisition Temperature (°C)** |
| --- | --- | --- | --- | --- |
| ***HvCesA1*** | TGTGGCATCAACTGCTAGGAAA | TGATAATCATCGCAAGACCG | 306 | 82 |
| ***HvCesA2*** | GCAAGTCAGTGGGCTTTAGTG | ACATGCTTAACGTAATTCAACAGA | 236 | 81 |
| ***HvCesA6*** | TTGTAGATGAAGACGGAAGGA | CATGCTTAACGTAATTCAACAGA | 280 | 82 |
| ***HvCesA4*** | GCCCAAGGGACCCATTCTTA | TGATAATCATCGCAAGACCG | 292 | 82 |
| ***HvCesA8*** | AGGACCATCAAGGGCGAAT | GTTTGAACGATCGGGGAAAT | 123 | 80 |

QPCR primers, product sizes and the optimal acquisition temperature for the various pMDC32/*HvCesA* constructs.

**Supplementary Figures**

**
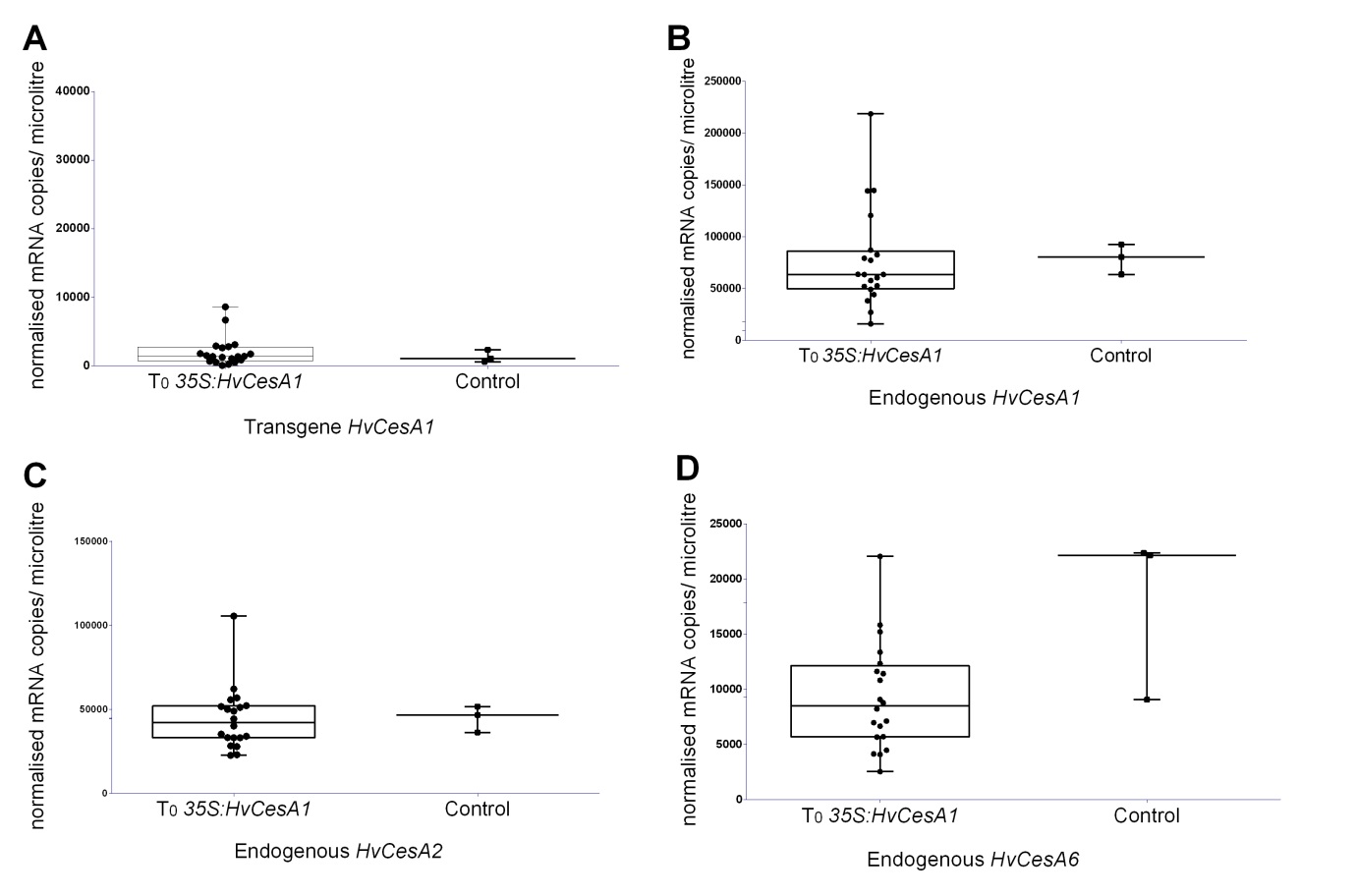
**

**Figure S1 Box plot graphs showing transcript profiles for all *35S:HvCesA1* plants**

The boundary of the box closest to zero indicates the 25th percentile, a line within the box marks the median, and the boundary of the box farthest from zero indicates the 75th percentile. Two ends of whiskers denote maximum and minimum value and each dot represents the mean transcript value per plant. (A) Transcript levels of the *HvCesA1* transgene were low, below the (B) corresponding *eHvCesA* transcript in control plants. For the same set of plants, endogenous transcript levels are shown in (B) for *eHvCesA1* (C) for *eHvCesA2* and (D) for *eHvCesA6*. Overall, except for *eHvCesA6*, averaged levels of *eHvCesA1* and *eHvCesA2* transcripts in transgenic plants were similar to levels measured in control plants.

**
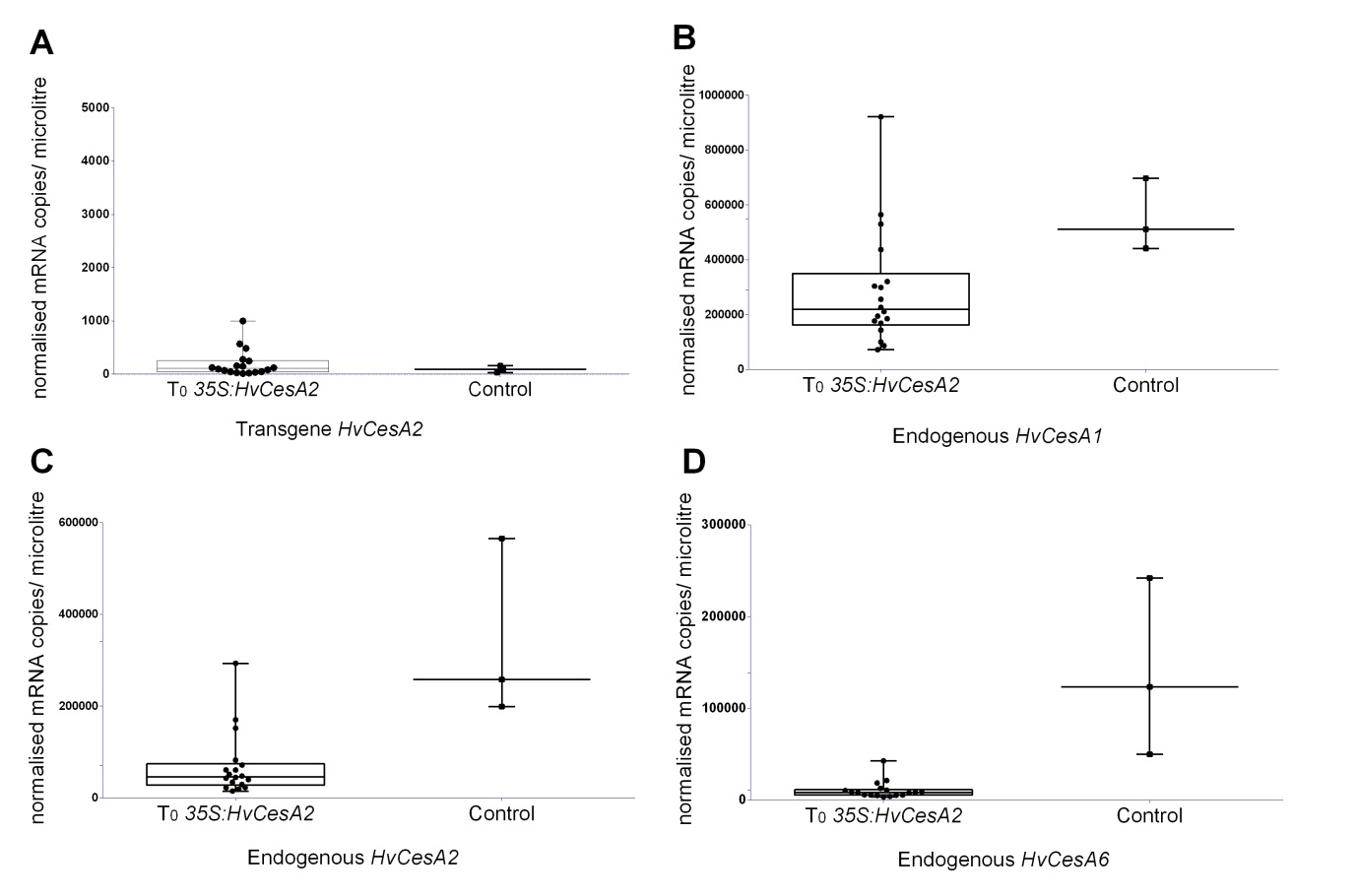
**

**Figure S2 Box plot graphs showing transcript profiles for all *35S:HvCesA2* plants**

The boundary of the box closest to zero indicates the 25th percentile, a line within the box marks the median, and the boundary of the box farthest from zero indicates the 75th percentile. Two ends of whiskers denote maximum and minimum value and each dot represents the mean transcript value per plant. (A) Transcript levels of the HvCesA2 transgene were low, below 10% of the corresponding *eHvCesA* transcript in control plants (C). For the same set of plants, corresponding endogenous transcript levels are shown in (B) for *eHvCesA1* (C) for *eHvCesA2* and (D) for *eHvCesA6*. For *35S:HvCesA2* plants, the transcript level for all endogenous *HvCesA* genes in control plants was on average much higher than in the transgenic set.

**
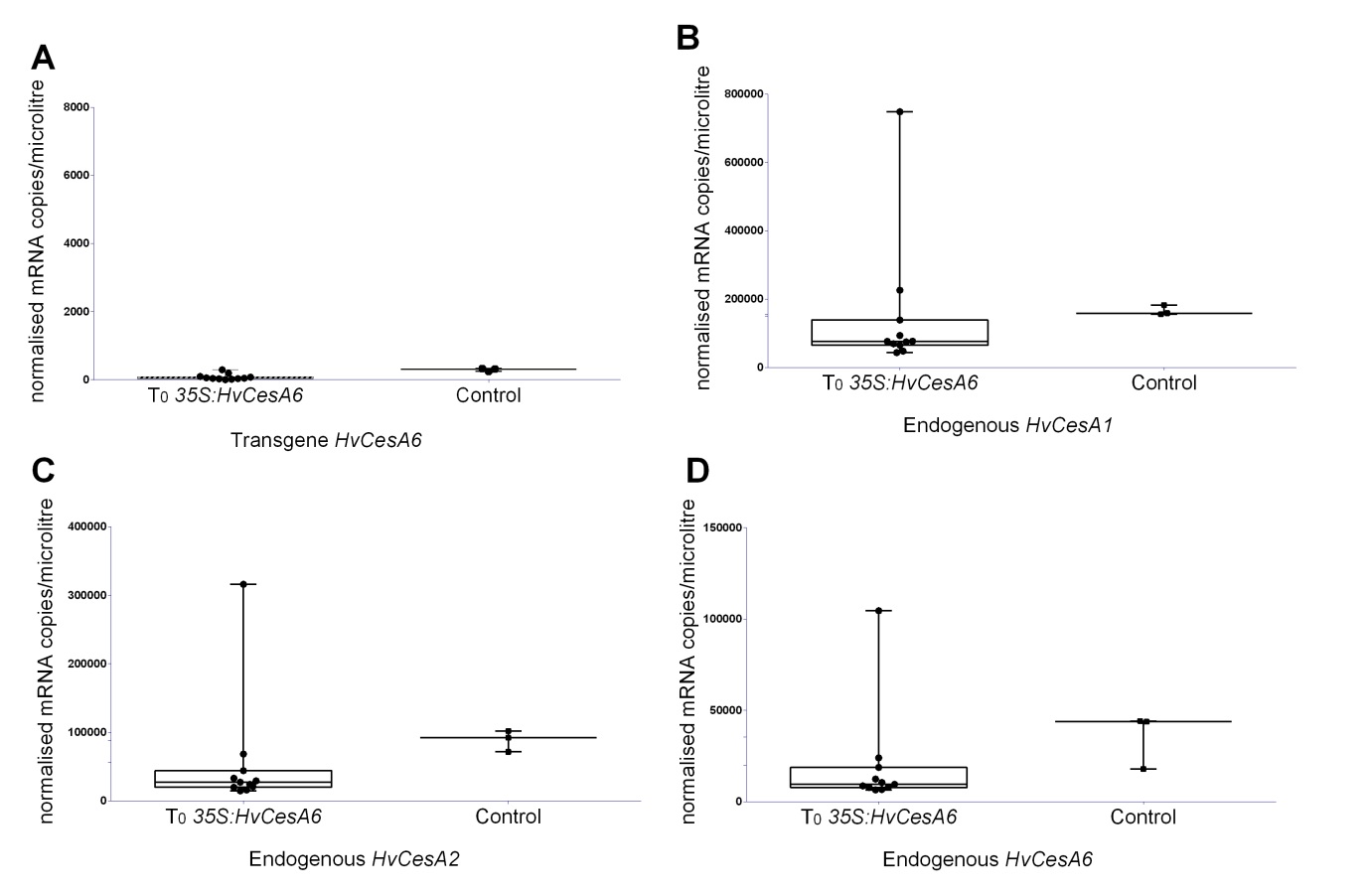
**

**Figure S3** Box plot graphs showing transcript profiles for all *35S:HvCesA6* plants

The boundary of the box closest to zero indicates the 25th percentile, a line within the box marks the median, and the boundary of the box farthest from zero indicates the 75th percentile. Two ends of whiskers denote maximum and minimum value and each dot represents the mean transcript value per plant. (A) Transcript levels of the HvCesA6 transgene were low, below 10% of the corresponding *eHvCesA* transcript in control plants. For the same set of plants, corresponding endogenous transcript levels are shown in (B) for *eHvCesA1* (C) for *eHvCesA2* and (D) for *eHvCesA6*. The majority of the endogenous transcript levels were below control levels.


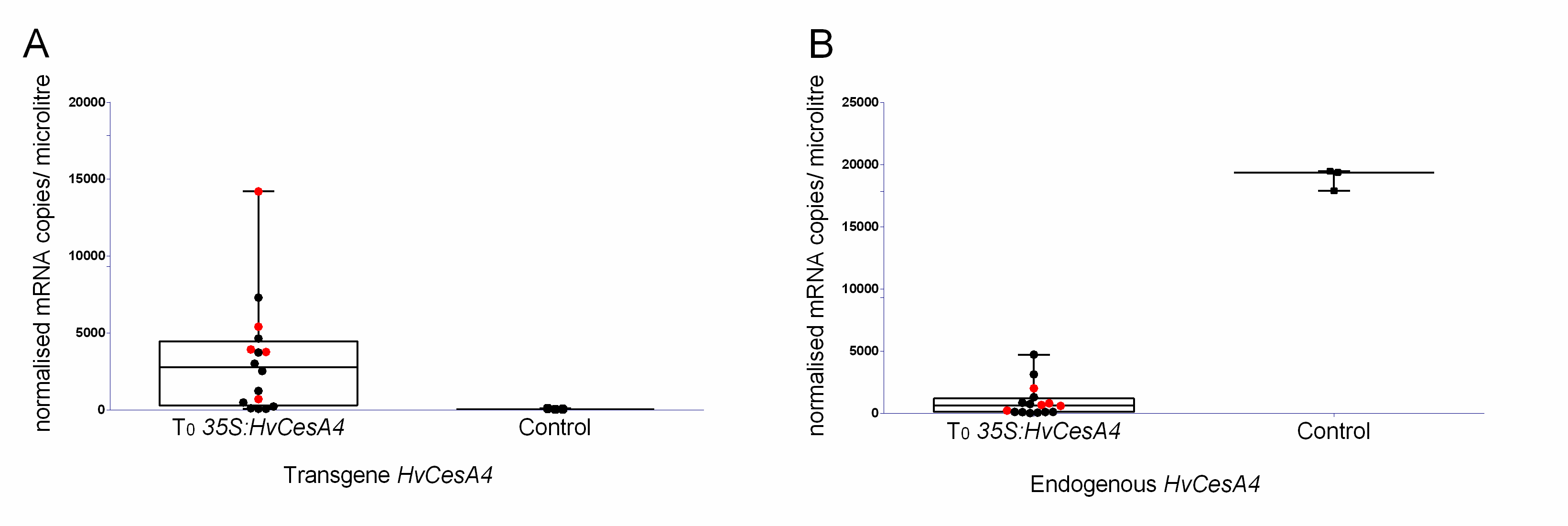


**Figure 5 Transcript abundance for transgene and endogenous genes in transgenic** ***35S:HvCesA4***. The boundary of the box closest to zero indicates the 25th percentile, a line within the box marks the median, and the boundary of the box farthest from zero indicates the 75th percentile. Two ends of whiskers denote maximum and minimum value and each dot represents the mean transcript value per plant. Transcript values for aberrant plants are marked in red. (A) Transcript levels of the *HvCesA4* transgene. For the same set of plants, corresponding endogenous transcript levels are shown in (B) for *eHvCesA4* and levels measured in three wild-type control plants.


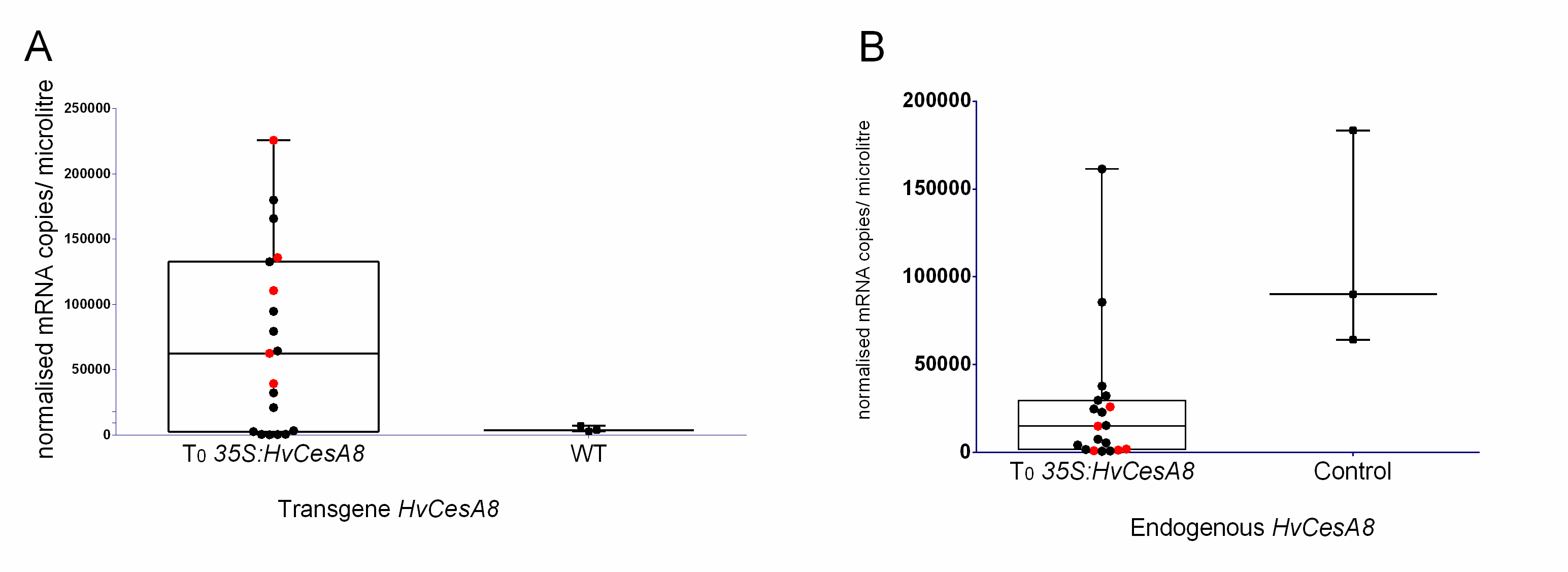


**Figure 6 Transcript abundance for transgene and endogenous genes in transgenic *35S:HvCesA8*.** The boundary of the box closest to zero indicates the 25th percentile, a line within the box marks the median, and the boundary of the box farthest from zero indicates the 75th percentile. Two ends of whiskers denote maximum and minimum value and each dot represents the mean transcript value per plant. Transcript values for aberrant plants are marked in red. (A) Transcript levels of the *HvCesA8* transgene. For the same set of plants, corresponding endogenous transcript levels are shown in (B) for *eHvCesA8* and levels measured in transgenic and three wild-type control plants.


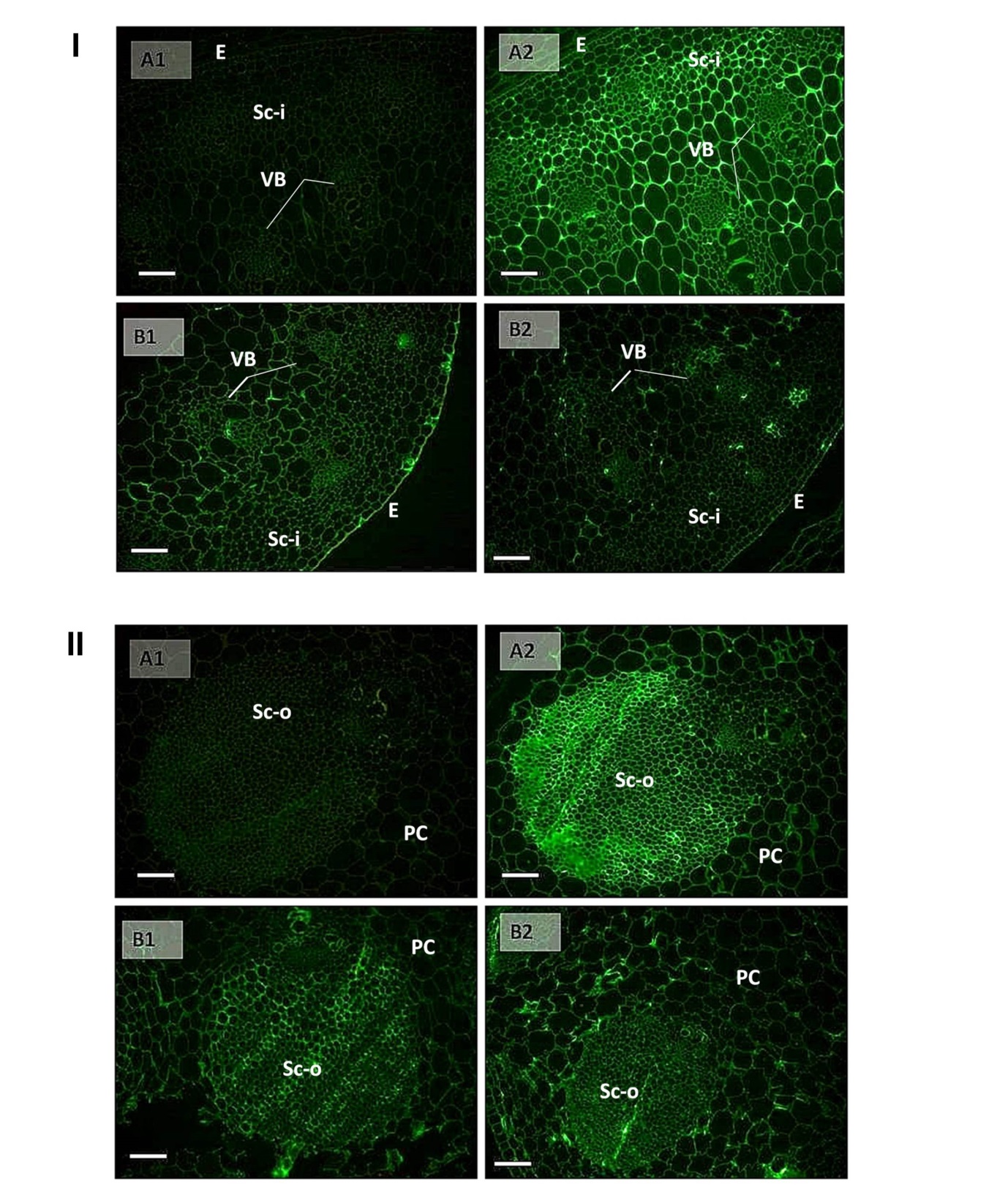


**Figure S6** Immunofluorescent labelling of T_2_ *35S:HvCesA4* node cross-sections.

Node sections were large and were observed as the inner stem layer (I) and the leaf sheath layer (II). For T_2_ *35S:HvCesA4* plants, fluorescence for both transgenic lines in all cell types was less intense in the inner stem layer but less so in leaf sheath layers when compared to controls. (A1) negative (same treatment as control and transgenic was applied but CBM3a was excluded), (A2) control= wild type or nulls, (B1) transgenic plant from Line 11 and (B2) transgenic plant from Line 15. Fluorescent images were taken at the same exposure and magnification for all samples. Scale bar is 100 µM. E=epidermis, VB= vascular bundle, PC= parenchyma cells, Sc-i = sclerenchyma cells in stem layer and Sc-o = sclerenchyma cells in leaf sheath layer.

**I**

**II**


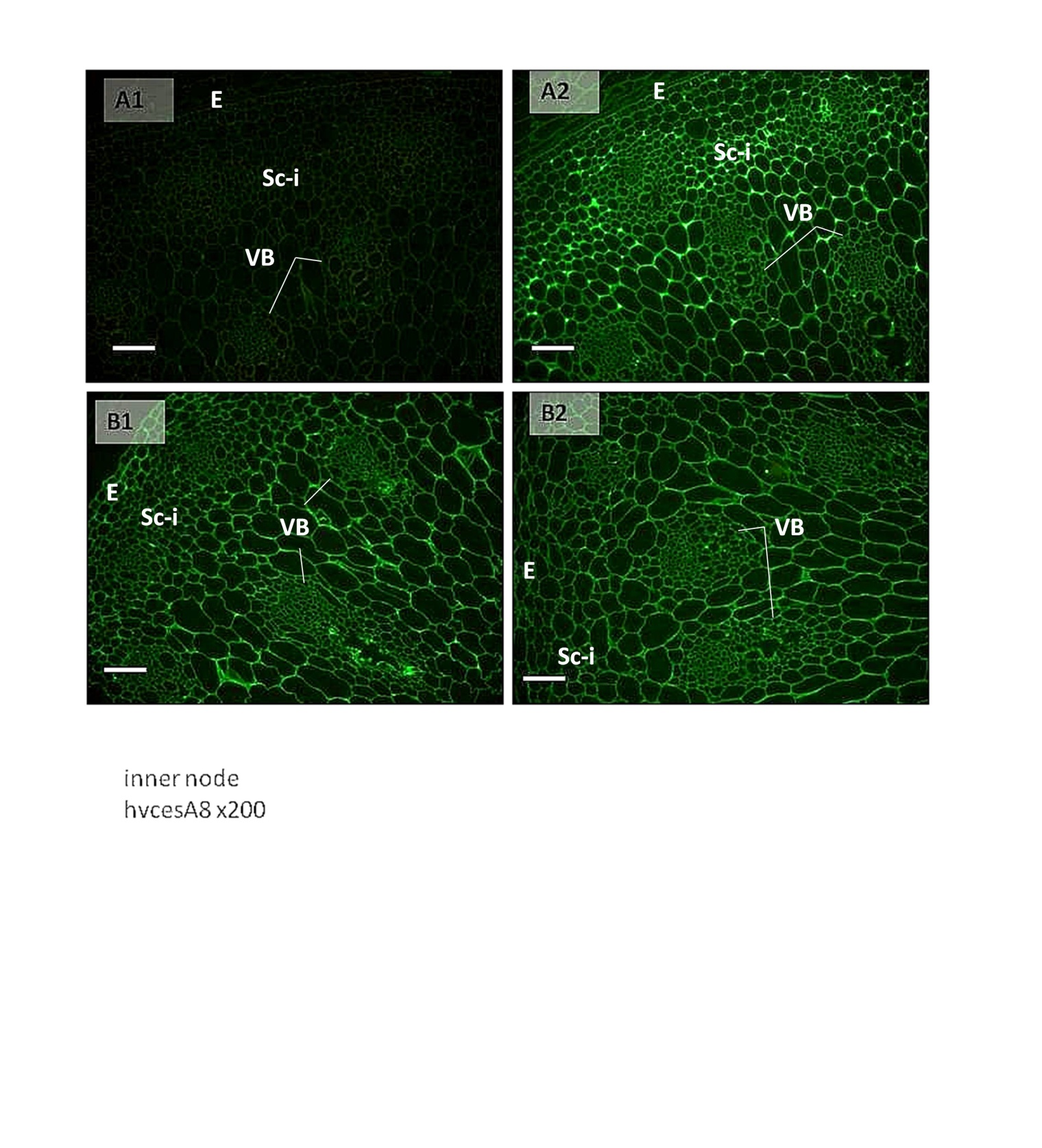


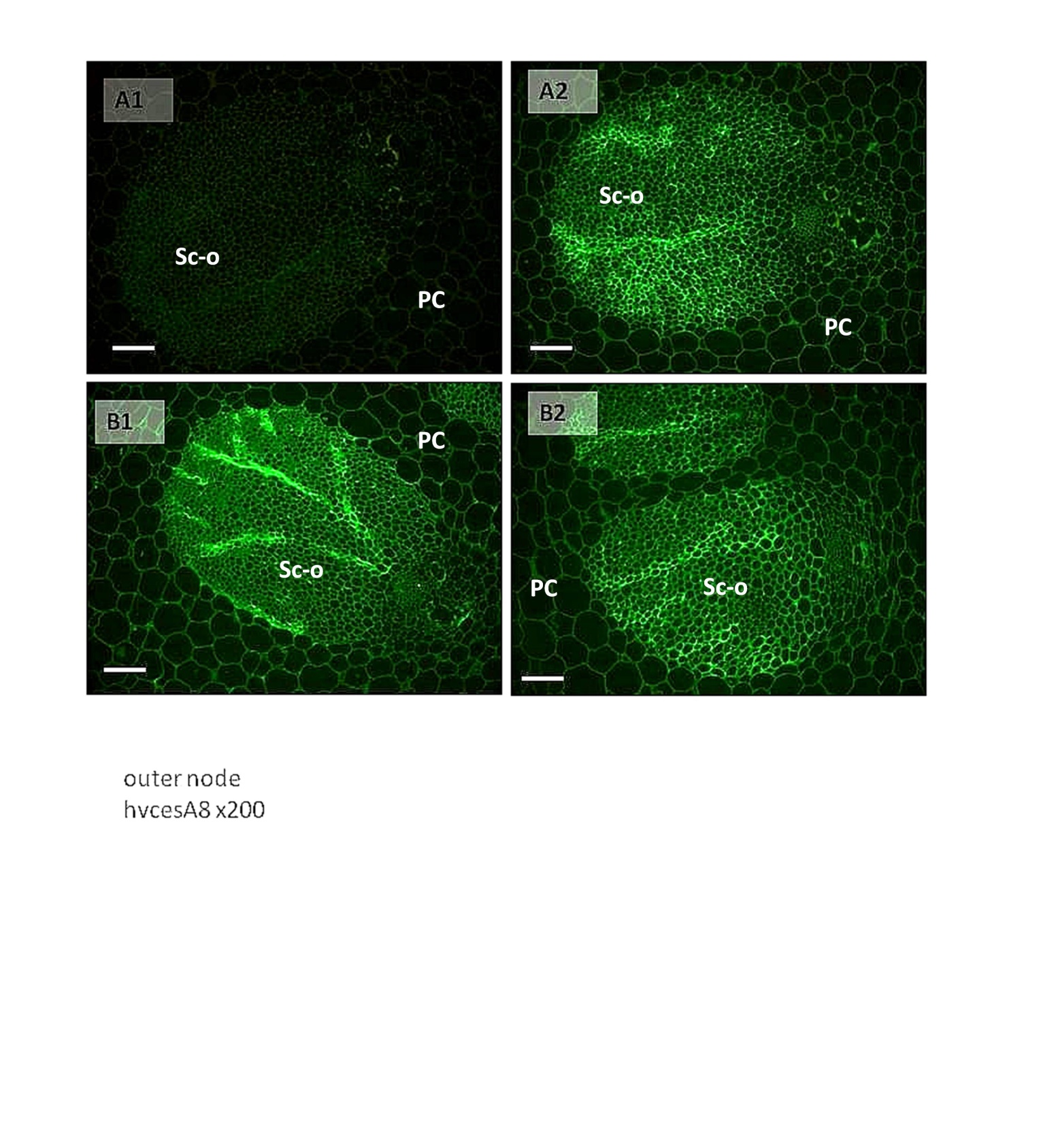


**Figure S7** Immunofluorescent labelling of T_2_ *35S:HvCesA8* node cross-sections.

For T_2_ *35S:HvCesA8* plants, no drastic difference between the fluorescence of transgenic and control plants was detected for any cell types in the node sections examined. (I) Inner stem layer and (II) Outer leaf sheath. (A1) negative (same treatment as control and transgenic was applied but CBM3a was excluded), (A2) control= wild type or nulls, (B1) transgenic plant from Line 11 and (B2) transgenic plant from Line 15. Fluorescent images were taken at the same exposure and magnification for all samples. Scale bar is 100 µM. E=epidermis, VB= vascular bundle, PC= parenchyma cells, Sc-i = sclerenchyma cells in stem layer and Sc-o = sclerenchyma cells in leaf sheath layer.

**
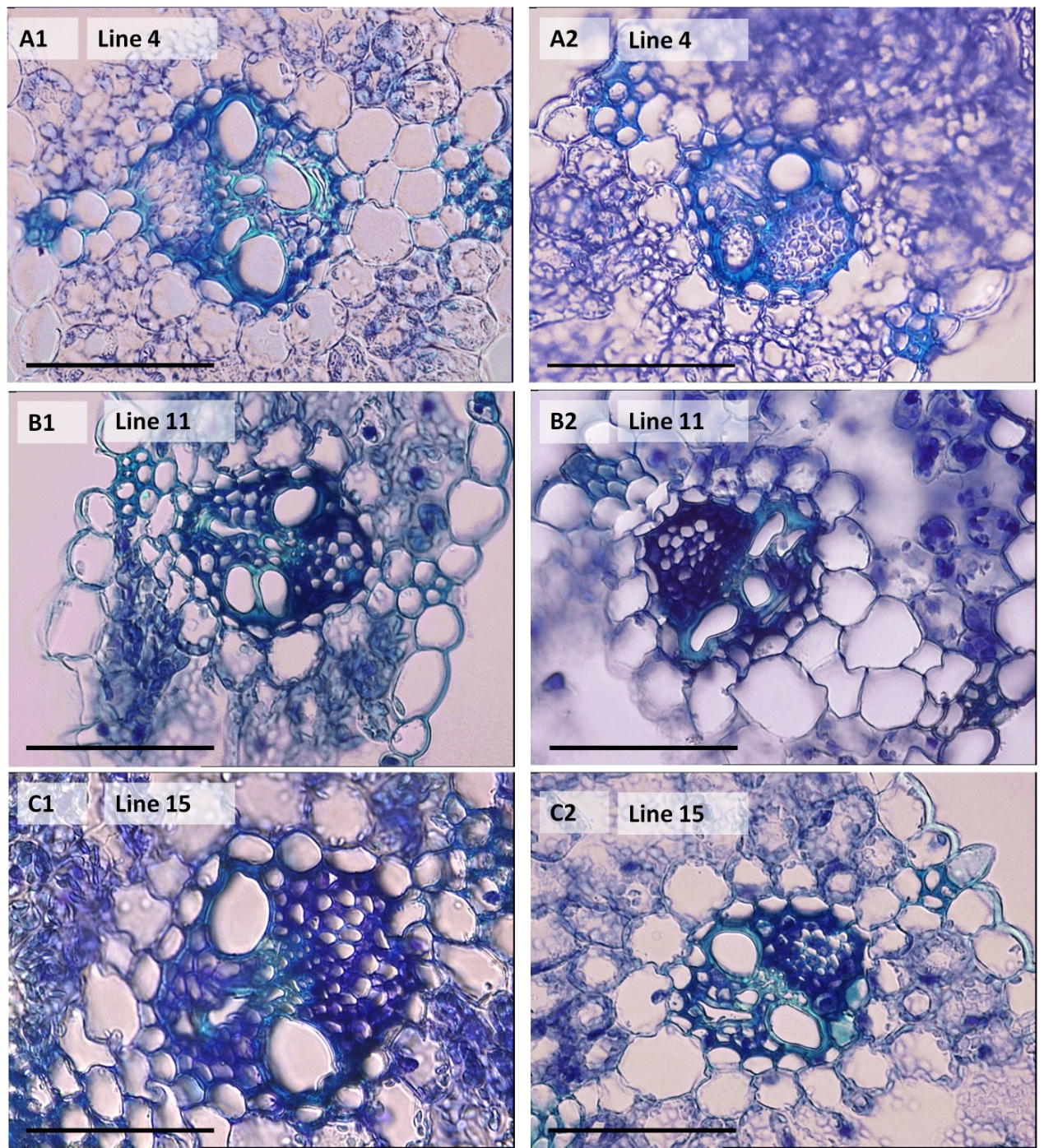
**

**Figure S8** Partially collapsed xylem vessels found in vibratome sections (30-50 µM) of leaves from *35S:HvCesA4*, T_1_ plants. All scale bars are 100 µM. (A1, A2), (B1, B2) and (C1,C2) are two plants from three sets of independent lines (line 4, line 11 and line 15) carrying the same construct.

*
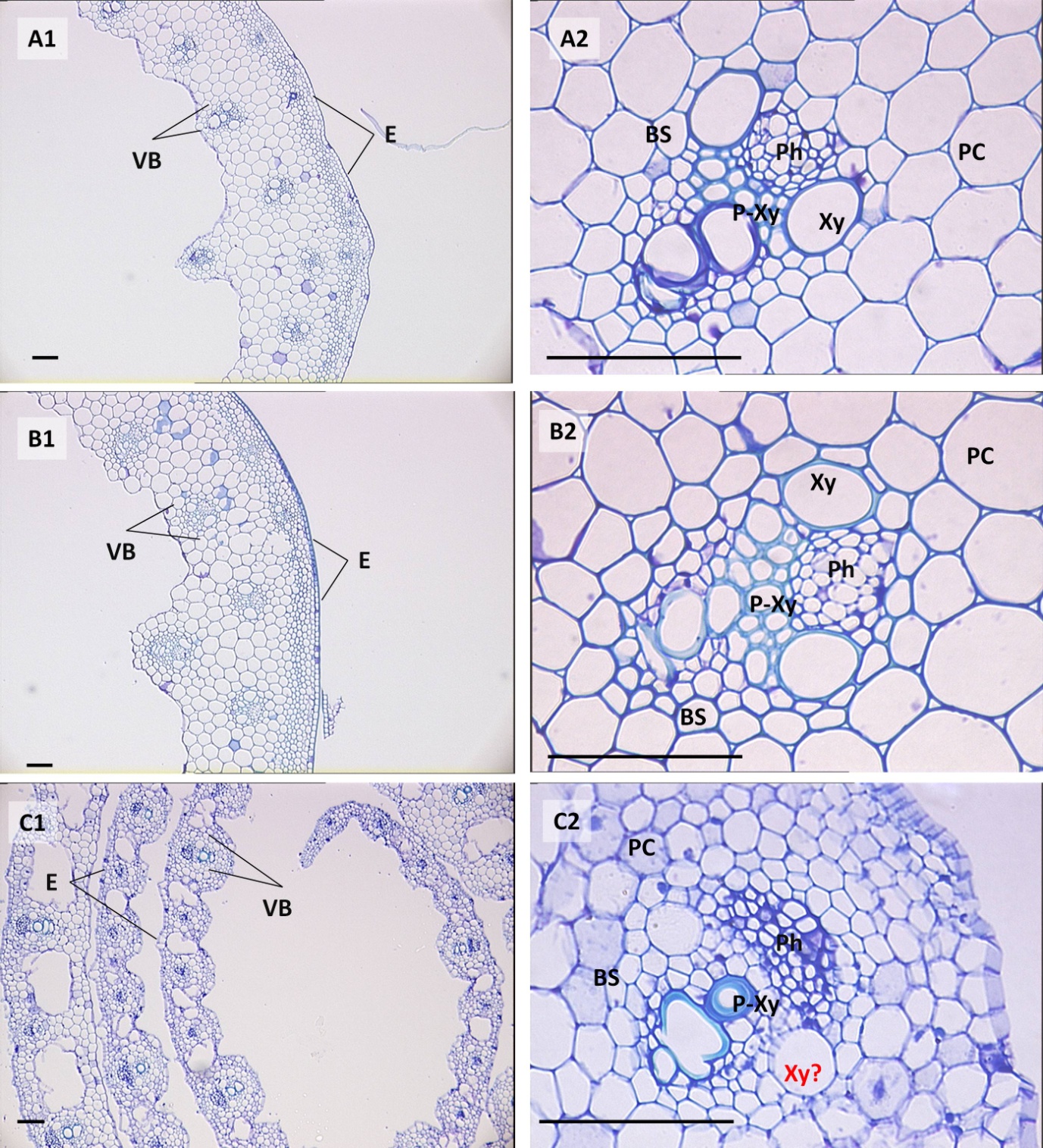
*

**Figure S9** Light microscopy of cross-sections of *35S:HvCesA8* stem internodes stained with Toluidine Blue.

(A) wild-type or null, (B) ‘brittle node” transgenic T_1_ plants and (C) severely stunted transgenic T_1_ plants. For (A) and (B) equivalent internodes were sectioned (~7 µM thick paraffin sections on the microtome). (C) These plants appeared to comprise only the leaves arising from the crown at the base of the plant. Numbers 1 or 2 indicate two different magnifications used to visualise the same sample. Scale bars denote 100 µM.

1= 100X Magnification, 2=630X Magnification of a vascular bundle. E=epidermis, VB= vascular bundle, Ph= phloem tissue, p-Xy=proto-xylem, Xy=meta-xylem, BS= bundle sheath, PC= parenchyma cells.


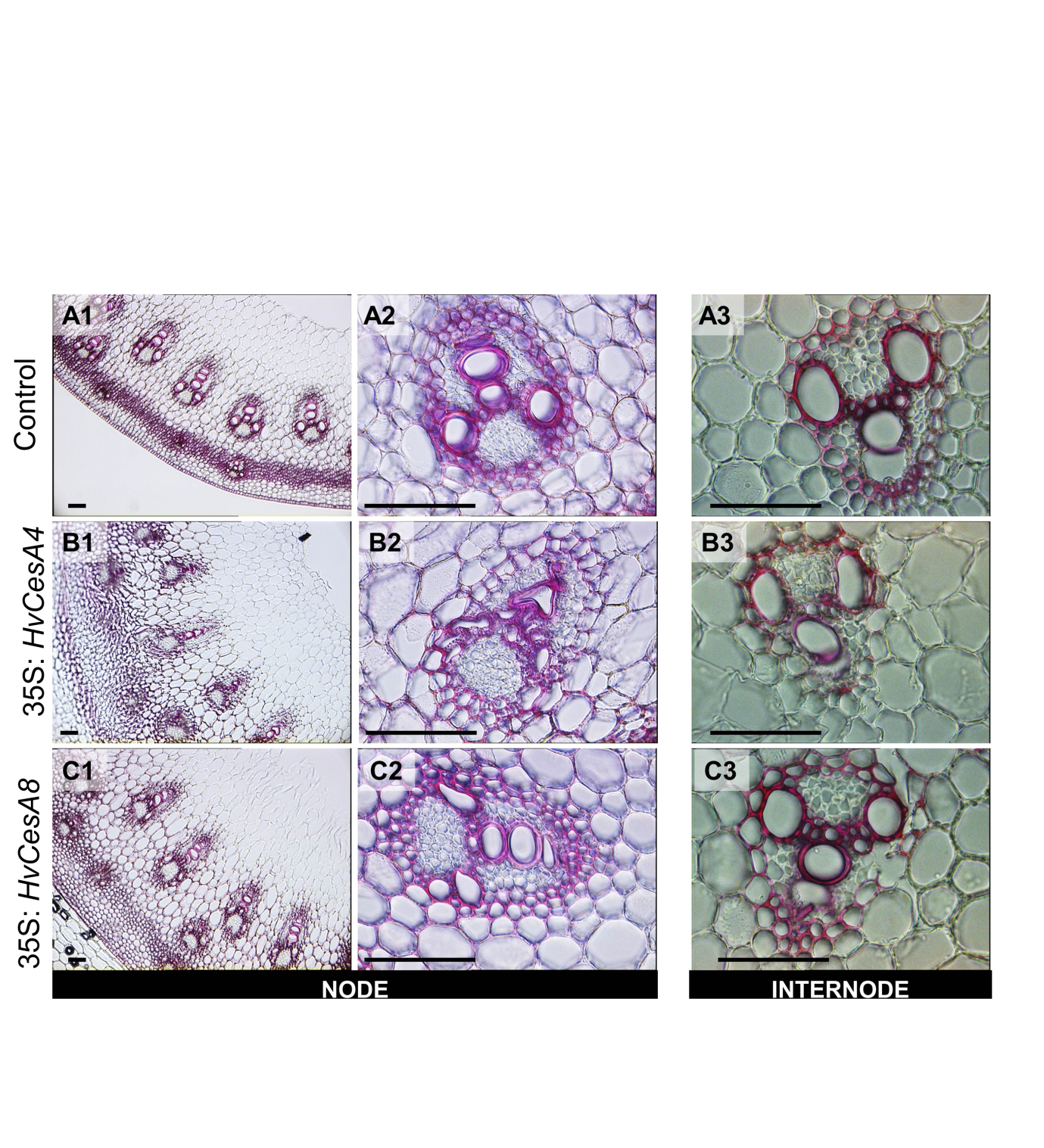


**Figure S10** Bright field microscopy of phloroglucinol-HCl stained node and internode cross-sections. (A) control= wild type or null plants, (B) T_2_ *35S:HvCesA4*, (C) T_2_ *35S:HvCesA8.* (1) node sections, (2) vascular bundle from node sections and (3) vascular bundle from the internode. Scale bars denote 100 µM. E=epidermis, VB= vascular bundle, Sc = sclerenchyma cells of inner stem layer, Ph = phloem tissue, Xy = xylem, BS= bundle sheath, PC= parenchyma cells.

CesA

3’UTR

CesA

NOS

3’UTR

Endogenous

Transgene

AAAAA

**Figure S11** Schematic representation of the primer binding sites for endogenous and transgene HvCesAs. Note that the 3’untranslated region (UTR) of transgene is shorter than the endogenous 3’UTR (~100bp), enabling a specific endogenous reverse primer to be designed (primer binding site absent in transgene). Primers specific for the transgene are designed with the reverse primer binding to the NOS terminator region found in the pMDC32 vector.
